# Don’t look, don’t think, just do it! Toward an understanding of alpha gating in a discrete aiming task

**DOI:** 10.1111/psyp.13298

**Published:** 2018-10-25

**Authors:** Germano Gallicchio, Christopher Ring

**Affiliations:** ^1^ School of Sport, Exercise & Rehabilitation Sciences University of Birmingham Edgbaston UK

**Keywords:** alpha gating, golf putting, neural efficiency, practice variability

## Abstract

Prior to and during movement, oscillatory alpha activity gates cognitive resources toward motor areas of the cortex by inhibiting neuronal excitability in nonmotor areas. The present study examined the effect of manipulating target variability on this alpha gating phenomenon. Using a baseline‐test‐retention design, we measured EEG alpha power, performance accuracy, and task difficulty in 32 recreational golfers as they putted golf balls (20 per target) to one central target (baseline, retention) and four targets of different directions and extents (manipulation). For participants in the random group (*n* = 16), target location varied with each repetition in a random fashion, whereas for participants in the blocked group (*n* = 16), it was kept constant within blocks. Regional analyses revealed a focal pattern of lower central alpha and higher occipital and temporal alpha. This topography was specific to preparation for movement and was associated with performance: smallest performance errors were preceded by decreased central combined with increased occipital alpha. The random group performed worse than the blocked group and found the task more difficult. Importantly, left temporal alpha prior to movement onset was lower for the random group than the blocked group. No group differences were found at baseline or retention. Our study proved that alpha gating can be altered by manipulating intertrial variability and thereby demonstrated the utility of the alpha gating model. Our findings underscore the importance of inhibiting occipital and left temporal areas when performing movements and provide further evidence that alpha gating reflects neural efficiency during motor tasks.

## 1 INTRODUCTION

The willful and repeated execution of an action induces a series of psychomotor adaptations consistent with increased efficiency (Brener, 1986). At the neurophysiological level, the brain becomes better at discriminating processes that are functional to optimal execution from those that are not; with repetition, the former are enhanced and the latter suppressed (Hatfield, 2018; Hatfield & Hillman, 2001). The execution of an action is characterized by a distinctive spatiotemporal pattern in the EEG, involving oscillatory activity within the alpha frequency (i.e., around 10 Hz) band. Specifically, prior to and during movement, alpha decreases over motor areas of the cortex while concurrently increasing over nonmotor areas (Neuper & Pfurtscheller, 2001; Pfurtscheller, 1992). The function of alpha is to exert inhibitory control across the cerebral cortex, whereby higher alpha indicates stronger neuronal inhibition and less alpha indicates greater release from inhibition (Klimesch, 2012; Klimesch, Sauseng, & Hanslmayr, 2007). Based on this evidence, the gating‐by‐inhibition model (Jensen & Mazaheri, 2010) proposes that neuronal excitability is diverted away from regions showing higher alpha power (i.e., greater inhibition) and routed toward regions showing lower alpha power (i.e., less inhibition). Consequently, the alpha gating model describes a mechanism that explains how the brain accomplishes the activation of motor areas alongside the inhibition of nonmotor areas to achieve psychomotor efficiency.

Research on skilled motor performance requiring precise motor control, such as target sports like golf putting and gun shooting, has revealed temporal and spatial dynamics of the alpha rhythm that account for inter‐ and intraindividual variations in performance and expertise (for review of studies, see Cooke, 2013; Hatfield, Haufler, Hung, & Spalding, 2004). Two recent studies have explored the utility of the alpha gating model as a framework to study the phenomenon of psychomotor efficiency. First, Gallicchio, Finkenzeller, Sattlecker, Lindinger, and Hoedlmoser (2016) examined alpha in a biathlon shooting task. They analyzed the topography of alpha in the second preceding each shot and described a focal pattern of simultaneous lower alpha in the central regions and higher alpha in temporal and occipital regions. Importantly, this pattern was associated with performance: lower central alpha and higher temporal alpha preceded improved shooting accuracy. Gallicchio and colleagues interpreted their findings as evidence that stronger alpha gating redirected neural resources more efficiently toward processes that supported performance and away from those unrelated to performance. Second, Gallicchio, Cooke, and Ring (2017) examined changes in alpha gating before and after practice of a golf putting task. They recorded alpha from recreational golfers as they putted balls to a hole before and after three training sessions. Their findings confirmed a shift in the topographical pattern that was consistent with the gating of resources away from temporal and occipital regions and toward central regions following motor learning. Importantly, they found that the largest improvements in performance following practice were associated with increased alpha power (indicative of greater inhibition) over the temporal regions.

The abovementioned studies provide preliminary evidence that a movement‐related alpha gate is associated with motor performance, and that its intensification—higher alpha in movement‐unrelated regions (i.e., temporal and occipital) and lower alpha in movement‐related regions (i.e., central)—can reflect improvements in psychomotor efficiency with practice. Accordingly, practicing a skill under conditions that strengthen the gating phenomenon may be expected to improve motor performance. However, there is currently no evidence that the movement‐related alpha gate can be modulated by the structure of the practice conditions. The present study was designed to fill this gap in our understanding of the alpha gating phenomenon and represents the first attempt to manipulate the strength of the alpha gate by varying the nature of the practice schedule. Specifically, we manipulated the trial‐by‐trial variability of the target in a golf putting task and compared the effects of variable practice and blocked practice (cf. Porter & Magill, 2010) on alpha oscillations. For some individuals the location of the target varied with each repetition in a random fashion, whereas for others it was kept constant within blocks of consecutive repetitions.

Repeating a movement under a schedule of random variability increases cognitive load during motor preparation by requiring that parameters, such as force and direction, are respecified for each movement (Lee, Magill, & Weeks, 1985) and by fostering comparisons of these parameters among the different movements (Shea & Morgan, 1979). If alpha gating reflects improved neural efficiency acquired through repetition, then random variability across repetitions should curb the development of psychomotor efficiency and interfere with the focal distribution of alpha across the cortex.

The aims of the present study were threefold. Our first study aim was to confirm the existence of the movement‐related alpha gating phenomenon and establish its behavioral relevance. Specifically, we expected to see a regional pattern of lower alpha power over central regions and higher alpha power over temporal and occipital regions that was specific to motor preparation. Furthermore, we expected that a stronger gate (i.e., higher alpha power over motor areas and lower alpha power over nonmotor areas) would be associated with better task performance.

Our second study aim was to examine the impact of target variability on motor performance, task difficulty, mental effort, and alpha gating. We hypothesized that, compared to blocked repetition, random repetition would result in worse performance, greater perceived difficulty and effort, and a weaker alpha gate (reflected in blunted enhancement of task‐relevant central regions and/or suppression of task‐irrelevant temporal and occipital regions).

Our third study aim was to evaluate the extent of any carryover effect of practicing a skill under a random repetition condition when reverting to a blocked repetition condition. Based on theories of motor learning arguing for enhanced performance following practice under a random, compared to a blocked, schedule (e.g., Schmidt, 1975), we expected that random practice compared to blocked practice would produce better performance and a stronger alpha gate (indicative of increased neural efficiency) at a blocked retention test.

## 2 METHOD

### 2.1 Participants

Thirty‐two right‐handed male recreational golfers were randomly allocated to a blocked practice group (age: *M* = 19.94, *SD* = 2.29 years; number of golf putting practice events in the last 12 months: *M* = 13.06, *SD* = 16.25) or to a random practice group (age: *M* = 20.25, *SD* = 1.81 years; number of golf putting practice events in the last 12 months: *M* = 11.06, *SD* = 9.01). None had a formal golf handicap. Participants were asked to refrain from alcohol, caffeine, and nicotine 3 hr prior to testing and were compensated with £10 and research credits. All provided signed consent to take part in the study. The study protocol was approved by the local research ethics committee.

### 2.2 Putting task

Participants putted golf balls (diameter 4.7 cm) with a blade‐style putter (length =91 cm) to a series of five targets—adhesive paper markers (diameter =0.6 cm)—positioned on a flat putting surface (turf tiles, length 5 m, width 1.5 m; Stimpmeter value: 2.27 m). One central target was at a distance of 2 m and in a straight line from the putting position. The four peripheral targets varied in terms of distance (far, near) and side (left, right). The two far and the two near targets were at distances of 2.5 and 1.5 m from the starting position, respectively. The two left and the two right targets were 0.15 m perpendicular to the line drawn from the ball to the middle target. The putting setup is illustrated in the online supporting information (Appendix S1).

Participants were instructed to putt at their own pace (i.e., with no time pressure) and as accurately as possible in order to “get the final position of the ball as close as possible to the target.” Prior to each putt, participants were instructed to stand in a relaxed position and maintain their gaze on a fixation cross placed at eyesight on the facing wall (c. 1.5 m away), until an acoustic tone (duration: 200 ms; frequency: 1,200 Hz) prompted them to look at a stimulus box. The box was positioned on the putting surface 15 cm away from the ball. The box informed them about the location of the upcoming target by illuminating one of five light‐emitting diodes (LEDs) for the duration of the trial. The arrangement of the five LEDs represented the spatial location of the targets on the putting surface. The tones and LEDs, which served as cue stimuli, were controlled by an Arduino Micro board (Arduino, Italy) interfaced with a computer running MATLAB (MathWorks, USA).

### 2.3 Procedure

Participants attended one 2‐hr session. After briefing and instrumentation for physiological recording, they performed 10 familiarization putts: one putt to each target; this sequence was repeated twice. Then, participants completed the putting task. They putted 120 balls in three conditions: baseline, one block of 20 putts; test, four blocks of 20 putts; and retention, one block of 20 putts. After each block, participants completed some self‐report measures assessing task difficulty and mental effort (see below for details).

In the baseline and retention conditions, the target was always the middle target. In the test condition, the target varied among the four peripheral targets, in either a blocked or random fashion according to group allocation. For participants in the blocked group, each putting block included only one of the four peripheral targets (i.e., 20 consecutive putts to the same target), and the sequence of blocks was randomized. For participants in the random group, each block included a pseudorandom sequence of the four peripheral targets; namely, all four targets were presented within each set of four consecutive putts, with the constraint that the same target could not be presented twice in a row. After each putt, the experimenter took a photograph of the target area using a ceiling‐mounted camera, repositioned the ball on the starting position, and then pressed a key to initiate the next trial. The time between the onset of the audiovisual cue and backswing initiation was 7.08 s (*SD* = 2.66 s) with a minimum of 3.04 s. The time between consecutive putts was 25.6 s (*SD* = 16.8 s), and the time between consecutive blocks was approximately 2 min. Upon completion of the putting task, the participant was debriefed and paid.

### 2.4 Physiological signals

Thirty‐two active electrodes were positioned on the scalp at Fp1, Fp2, AF3, AF4, F7, F3, Fz, F4, F8, FC5, FC1, FC2, FC6, T7, C3, Cz, C4, T8, CP5, CP1, CP2, CP6, P7, P3, Pz, P4, P8, PO3, PO4, O1, Oz, O2 (10–20 system, Jasper, 1958) to record the EEG. Two active electrodes were positioned on each mastoid. Four active electrodes were placed at the outer canthus and below each eye to record horizontal and vertical electrooculogram (EOG). And finally, two electrodes were placed on the right clavicle and left lower ribs to record the electrocardiogram (ECG; chest‐configuration Lead II montage). All channels were recorded in monopolar. Common mode sense and driven right leg electrodes were used to enhance the common mode rejection ratio of the signal. The signal was amplified and digitized at 2048 Hz with 24‐bit resolution, with no online filter, using the ActiveTwo recording system (Biosemi, The Netherlands).

Digital triggers were sent to the BioSemi system to identify the onset of the audiovisual cue onset and backswing initiation, identified by the putter head being moved away and thereby breaking from an infrared beam controlled by a digital switch (E18‐D80NK). In addition, vibrations from putter‐ball impact were recorded using a piezo sensor (MiniSense 100) attached to the back of the putter head and interfaced with the BioSemi system as an external analog channel. Offline, a bespoke MATLAB script identified the timing of cue, backswing, and impact events for each putt.

All physiological channels were referenced to the mastoids, downsampled to 512 Hz, and band‐pass filtered 0.1–40 Hz (FIR [finite impulse response], filter order = 2^15. Epochs were cut from −3.5 to +1.5 s relative to backswing initiation and cue, and then voltages were centered within each epoch (i.e., the epoch mean was subtracted from each data point in that epoch). Epochs were visually inspected, and those showing movement artifacts were discarded (these trials were also discarded from other non‐EEG analyses). The number of backswing‐centered epochs that were retained was 19.84 (*SD* = 0.37, minimum = 19) for the baseline condition, 79.25 (*SD* = 2.66, minimum = 65) for the test condition, and 19.91 (*SD* = 0.30, minimum = 19) for retention. The number of cue‐centered epochs that were retained was 19.88 (*SD* = 0.34, minimum = 19) for the baseline condition, 79.21 (*SD* = 2.66, minimum = 65) for the test condition, and 19.91 (*SD* = 0.30, minimum = 19) for retention. No bad channels were identified. Independent component analysis (ICA) weights were obtained through the RunICA infomax algorithm (Makeig, Bell, Jung, & Sejnowski, 1996) running on EEG, EOG, and ECG signals (i.e., 38 channels yielding same number of independent components) that, prior to ICA, were downsampled to 256 Hz, 2–40 Hz band‐pass filtered (FIR, filter order = 1,000), and concatenated across all conditions within each participant. Then, ICA weights were applied to the 0.1–40 Hz filtered signals, and the components that presented obvious nonneural activity upon visual inspection (e.g., eyeblinks, horizontal eye movements, cardiac artifact, muscle/movement artifacts) were manually rejected. On average, 4.94 components (*SD* = 1.39) were rejected per participant. Finally, ECG and EOG channels were discarded, and the remaining 32 EEG channels were average referenced. These preprocessing steps were performed using EEGLAB functions (Delorme & Makeig, 2004) for MATLAB.

### 2.5 Measures

#### 2.5.1 Performance

Performance on each putt was scored as radial error (cm), length absolute error (cm; hereafter length error), and angle absolute error (degrees; hereafter angle error). A trial was discarded from any further analysis when the ball rolled off of the putting surface (this occurred only once). A camera system with bespoke MATLAB scripts, inspired by Neumann and Thomas (2008), was used to score performance (see supporting information Appendix S1).

#### 2.5.2 Self‐report

Task difficulty was measured by asking each participant to rate “How difficult was it to get the ball to finish within 5 cm of the target(s)?” on a Likert scale ranging from 1 (*not difficult at all*) to 7 (*extremely difficult*). Mental effort was measured by asking each participant to rate “How much mental effort did you exert while putting?” on a Likert scale ranging from 1 (*no effort at all*) to 7 (*extreme effort*). Both items were rated after each block of 20 putts in relation to the block of putts just completed.

#### 2.5.3 Alpha power

**Figure 1:**
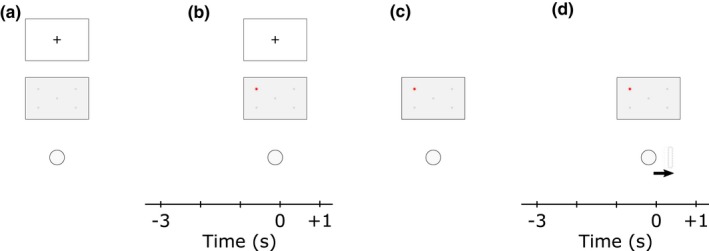
EEG epoching relative to the movements involved in the putting task. (a) The participant stood upright in front of the ball and maintained his gaze on the fixation cross located at eyesight on the opposite wall. (b) Cue onset: one of the LEDs turned on concomitantly to the acoustic tone, informing the participant of the location of the target. (c) At his own time, the participant positioned the putter head next to the ball and prepared for the putt. (d) Backswing initiation: the participant initiated the backswing. EEG alpha was examined from −3 to +1 s relative to this instant

Time‐frequency decomposition was performed through short‐time fast Fourier transform (FFT) conducted on 65 overlapping windows, each of 1 s, with central points ranging from −3 to 1, relative to backswing onset and cue onset (Figure 1). Prior to FFT, data points in each window were Hanning tapered and zero padded to reach 4 s. This procedure generated complex‐valued coefficients in the time‐frequency plane with a precision of 0.06 s and 0.25 Hz, separately for each channel and trial. Signal amplitude was doubled for all positive frequencies, and alpha power was computed as the squared amplitude in the 8–12 Hz frequency range.

**Figure 2:**
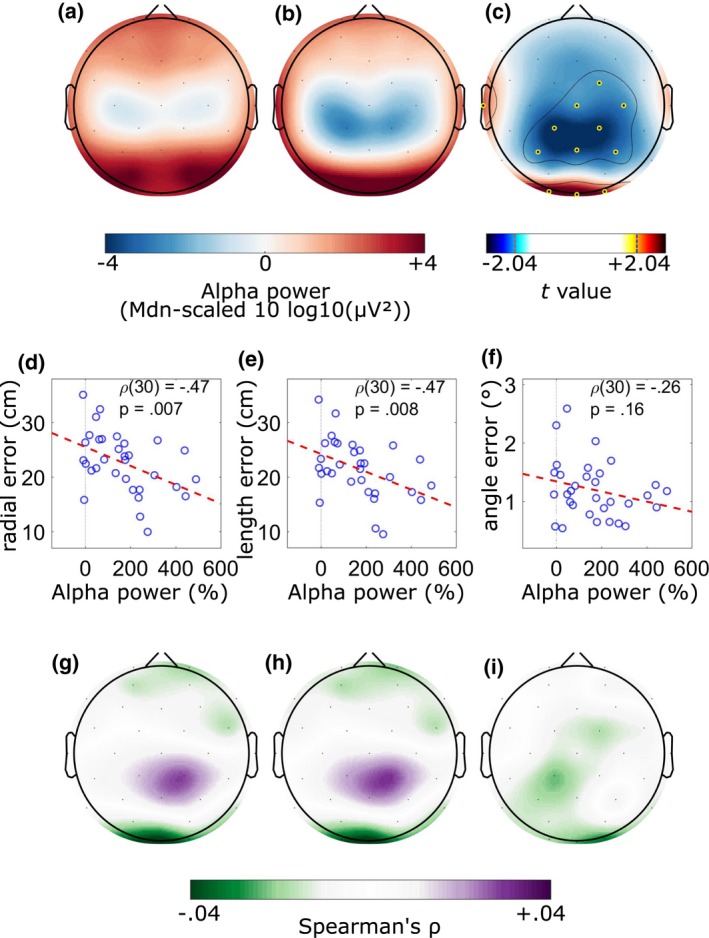
Scalp maps of absolute alpha power in the 2 s prior to (a) cue onset and (b) backswing initiation. (c) Scalp map of paired samples *t* values (*df* = 31) comparing precue and prebackswing alpha power. Values of −2.04 and 2.04 correspond to *p* = 0.05 on a *t* distribution with 31. Statistical thresholding was applied using the maximum‐statistic permutation testing (Cohen, 2014; Nichols & Holmes, 2002) controlling for multiple comparisons in the channel dimension with alpha set at 0.01. Statistically significant channels are indicated by yellow‐black markers and are surrounded by a solid contour line. Scatterplots showing (d) radial, (e) length, and (f) angle errors as a function of relative alpha power at Oz in the 2 s prior to backswing initiation along with Spearman's ρ and *p* values. Scalp maps of Spearman's ρ computed between relative alpha power in the 2 s prior to backswing initiation and (g) radial error, (h) length error, and (i) angle error. Statistical thresholding was applied using the maximum‐statistic permutation testing controlling for multiple comparisons in the channel dimension with alpha set at 0.01: no significant effect was revealed. All participants (*n* = 32) and only trials from the baseline condition were included in these graphs

We computed two metrics of alpha power per trial. Absolute alpha power was computed with no baseline correction; instead, skewness and between‐subjects differences in the power density distribution were minimized through a median‐scaled log transformation, whereby power values of each participant were scaled by the median of all values within that participant and then subjected to a 10·log10 transformation (cf. Gallicchio, Finkenzeller, et al., 2016). Absolute alpha power was computed for both types of epoch—time‐locked to backswing initiation and cue onset. Relative alpha power was computed only for the epoch that was time‐locked to backswing initiation as percentage change from a baseline, identified as the 2 s preceding cue onset (averaged across trials, separately per each condition) using the formula described in Pfurtscheller and Lopes da Silva (1999): $r_{t,p,c}=100\left(b_{t,p,c}-\;{\overset{¯}{c}}_{c}\right)/{\overset{¯}{c}}_{c}$r_{{t,p,c}} = 100\left( {b_{{t,p,c}} ‐ ~\bar{c}_{c} } \right)/\bar{c}_{c}, where *r_t,p,c_* indicates relative alpha power at time *t*, putt *p*, and condition *c* (i.e., baseline, test, retention), *b_t,p,c_* indicates alpha power time‐locked to backswing initiation at time *t*, putt *p*, and condition *c*, and $\overset{¯}{c}$\bar{c} indicates alpha power averaged across the data points within the 2 s preceding cue onset and across putts in condition *c*. Six regions of interest (ROI) were identified based on inspection of topographic maps (Figures 1b, 2a): frontal (F3, Fz, F4), left temporal (T7, F7, CP5), left central (C3, CP1), right central (C4, CP2), right temporal (T8, F8, CP6), and occipital (O1, Oz, O2). Values within each ROI were averaged. Signal processing was performed in MATLAB.

### 2.6 Statistical analyses

#### 2.6.1 Alpha gating

A 6 ROI × 2 Epoch Type (cue, backswing) analysis of variance (ANOVA) on absolute alpha power was conducted to evaluate the presence of regional effects of alpha power consistent with the gating phenomenon and, by comparing the 2 s preceding cue onset against the 2 s preceding backswing initiation, to determine whether this regional phenomenon was specific to preparation for putting. Additionally, we explored regional effects through nonparametric permutation testing. The multiple comparison problem (i.e., one test for each channel) was solved through the “maximum statistic” method (Cohen, 2014; Nichols & Holmes, 2002) applied to the channel dimension. Namely, we compared paired samples *t* values of each channel with an empirical distribution of *t* values constructed in the following way. First, we permuted the data by randomly swapping the *cue* and *backswing* labels within each participant. Second, we ran a paired samples *t* test separately for each channel. Third, we pooled the *t* values across channels and stored the two most extreme values (i.e., minimum and maximum). Fourth, we repeated this procedure 1,000 times to create a distribution of 2,000 minimum and maximum *t* values. Finally, we compared the nonpermuted *t* value of each channel with the empirical distribution of *t* values described above: we computed *p* values as the proportion of the permutation *t* values that were more extreme than the *t* value of each channel.

Spearman's rank correlations (ρ) explored the association between performance and relative alpha power in the 2 s preceding backswing initiation. These analyses were conducted on the putts from the baseline condition, pooling participants from both groups (*n* = 32). In addition, we explored the topography of the alpha‐performance correlation through permutation testing corrected for multiple comparisons across the channel dimension. Namely, the ρ coefficient obtained for each channel was compared with a distribution of the 2,000 most extreme ρ coefficients across channels (i.e., 1,000 minimum and 1,000 maximum), whereby at each repetition participants were permuted for one of the two variables involved in the correlation.

#### 2.6.2 Manipulation of target variability

These analyses evaluated the impact of the manipulation of target variability on performance, self‐report, and alpha power measures. Independent samples *t* tests examined group differences in performance and self‐report measures of difficulty and mental effort (as change scores relative to baseline) on trials of the test condition. Performance absolute scores are reported in Appendix S2.

A 2 Group × 6 ROI × 4 Time × 4 Subset ANOVA was conducted on relative alpha power (i.e., as percentage change from the 2 s preceding cue onset). The time factor identified four 1‐s intervals from −3 to +1 relative to backswing initiation. The subset factor identified four 20‐putt sets, so that Subset 1 included the first five putts struck to each target—these were consecutive putts for the blocked but not the random group—Subset 2 included the next five putts to each target, and so on. Only trials from the test condition were included in these analyses. Additionally, we explored the topography of group differences through permutation testing controlling for multiple comparisons in the Channel × Time dimensions, separately for each subset. Namely, independent samples *t* values computed for each channel and each time were compared with a distribution of the 2,000 most extreme *t* values across channels and time (i.e., 1,000 minimum and 1,000 maximum) whereby group allocation was permuted across participants at each repetition.

#### 2.6.3 Retention effects

These analyses examined the short‐term persistence of effects following the manipulation of target variability. Group differences in performance and self‐report measures of difficulty and mental effort (expressed as change scores relative to baseline) were assessed through independent samples *t* tests. A 2 Group × 6 ROI × 4 Time ANOVA was conducted on relative alpha power. Only trials from the retention condition were considered in these analyses. Additionally, we explored the topography of group differences through permutation testing controlling for multiple comparisons in the Channel × Time dimensions with the same procedure described for the analyses of target variability.

#### 2.6.4 Additional frequency bands

In order to evaluate the involvement of frequency bands other than alpha, we conducted the analyses described above in the theta (4–6 Hz) and beta (15–25 Hz) frequency bands. In addition, to explore patterns within the broad alpha band, we analyzed separately the lower (8–10 Hz) and upper (10–12 Hz) alpha sub‐bands. These supplementing analyses are reported in Appendix S4.

The multivariate solution was adopted where appropriate (Vasey & Thayer, 1987) and Wilks’ lambda (λ) reported. Significant interactions were interrogated using post hoc *t* tests and polynomial trend analyses. Univariate partial eta‐squared ($\eta_{p}^{2}$) and *r* ^2^ were reported as measures of effect size, with values of 0.02, 0.13, and 0.26 reflecting small, medium, and large effects, respectively (Cohen, 1992).

## 3 RESULTS

### 3.1 Alpha gating

To address our first study aim, we conducted analyses to ascertain the existence of the movement‐related alpha gating phenomenon and its behavioral relevance. The regional distribution of absolute alpha power is shown as a function of epoch type in Figure 2a,b. The ROI × Epoch Type ANOVA yielded a main effect of ROI, *F*(5, 27) = 157.95, *p* < 0.001, λ = 0.033, $\eta_{p}^{2}$ = 0.889, indicating that alpha power was highest for the occipital region, lower for the left and right temporal and frontal regions, and lowest for the left and right central regions. This effect was superseded by a ROI × Epoch interaction, *F*(5, 27) = 47.17, *p* < 0.001, λ = 0.103, $\eta_{p}^{2}$ = 0.767: post hoc paired samples *t* tests revealed that alpha power decreased from precue to prebackswing for the left central, right central, and frontal regions, *t*s(31)>2.90, *p*s <0.007, and increased for the occipital region, *t*(31) = 7.50, *p* < 0.001. No differences emerged for left, *t*(31) = 0.70, *p* = 0.49, and right, *t*(31) = −0.84, *p* = 0.41, temporal regions. No main effect emerged for epoch type, *F*(1, 31) = 0.46, *p* = 0.50, $\eta_{p}^{2}$ = 0.015. Channel‐wise exploratory analyses conducted through permutation testing revealed that, compared to the precue period, prebackswing alpha power decreased for central channels (FC2, Cz, C4, CP1, CP2, P3, Pz, P4) and increased for occipital (O1, Oz, O2) and left temporal (T7) channels (Figure 2c).

To rule out the existence of group differences at baseline, we conducted a 2 Group × 6 ROI × 4 Time ANOVA on relative alpha power (i.e., alpha power around backswing initiation as percentage change from the 2 s preceding cue onset). The results of this analysis are reported fully in Appendix S3, along with exploratory channel‐wise analyses of group differences through permutation testing. No group effects were revealed at baseline.

Channel‐wise Spearman's correlations between relative alpha power and radial error revealed that the participants who obtained lower radial error were those that showed the largest increase in alpha power at Oz, ρ(30) = −0.47, *p* = 0.007, and O1, ρ(30) = −0.38, *p* = 0.04, compared to the precue onset period. Similar effects were revealed for length error (Oz: ρ(30) = −0.47, *p* = 0.008; O1: ρ(30) = −0.36, *p* = 0.04). No effects were revealed for angle error. Figure 2 illustrates the scatterplots of the relation between relative alpha power at Oz and radial error (panel d), length error (panel e), and angle error (panel f). Figure 2g,h,i show the topography of these correlations together with the outcome of permutation testing corrected for multiple comparisons. These analyses indicated that the findings reported above did not survive a rigorous control of multiple testing. However, the exploration of multiple frequency bands (i.e., theta, lower alpha, upper alpha, and beta) indicated that these correlations emerged only for the alpha band and particularly for the upper alpha sub‐band (see Appendix S4).

### 3.2 Manipulation of target variability

To address our second study aim, we conducted analyses to examine the impact of the manipulation of target variability on performance, self‐reported difficulty, and mental effort, and alpha gating.

#### 3.2.1 Performance

Compared to baseline putts, radial error decreased for the blocked group (*M* = –2.59, *SD* = 4.33 cm) and increased for the random group (*M* = 0.66, *SD* = 4.40 cm), which produced a significant group difference in terminal distance from the target, *t*(30) = −2.11, *p* = 0.04, *r* ^2^ = 0.359. Length error decreased in both the blocked (*M* = −3.02, *SD* = 4.41 cm) and random (*M* = −0.23, *SD* = 4.46 cm) groups, *t*(30) = −1.78, *p* = 0.09, *r* ^2^ = 0.309. Angle error increased for both the blocked (*M* = 0.22, *SD* = 0.43 degrees) and random (*M* = 0.52, *SD* = 0.48 degrees) groups, *t*(30) = −1.90, *p* = 0.07, *r* ^2^ = 0.328. Potential group differences in putting performance were further explored through independent samples *t* tests conducted separately for each subset of putts; where subset refers to the first, second, third, and fourth set of five putts struck to each target (i.e., 20 putts per each subset). These analyses revealed that clear group differences in extent‐based errors emerged during the second subset of putts and that these differences faded during the third and fourth subset of putts (Table 1).

**Table 1:** Mean (*SD*) of performance measures of each group as change scores from the baseline condition in each subset and results of independent samples *t* tests

Performance measure	Blocked	Random	*t*(30)	*p*	*r* ^2^
Subset 1					
**Δ** radial error (cm)	0.37 (6.78)	2.87 (5.51)	−1.15	0.26	.215
**Δ** length error (cm)	0.06 (7.37)	1.83 (5.74)	−0.76	0.46	.137
**Δ** angle error (degrees)	0.27 (0.62)	0.63 (0.61)	−1.67	0.11	.292
Subset 2					
**Δ** radial error (cm)	−2.65 (3.59)	1.69 (3.47)	−3.47	0.002	.535
**Δ** length error (cm)	−3.05 (3.89)	0.91 (3.47)	−3.03	0.01	.484
**Δ** angle error (degrees)	0.18 (0.43)	0.54 (0.46)	−2.32	0.03	.390
Subset 3					
**Δ** radial error (cm)	−4.04 (5.24)	−0.68 (5.37)	−1.79	0.08	.311
**Δ** length error (cm)	−4.55 (5.3)	−1.25 (5.23)	−1.77	0.09	.307
**Δ** angle error (degrees)	0.23 (0.48)	0.36 (0.49)	−0.76	0.46	.137
Subset 4					
**Δ** radial error (cm)	−4.01 (4.99)	−1.21 (6.04)	−1.43	0.16	.253
**Δ** length error (cm)	−4.48 (5.07)	−2.38 (6.25)	−1.04	0.31	.187
**Δ** angle error (degrees)	0.20 (0.56)	0.56 (0.61)	−1.74	0.09	.303

Note

A negative change score indicates that performance improved during the test compared to baseline.

#### 3.2.2 Self‐report

Compared to the baseline condition, self‐reported difficulty increased more for the random group (*M* = 0.97, *SD* = 1.15) than the blocked group (*M* = 0.11, *SD* = 1.06), *t*(30) = −2.20, *p* = 0.04, *r* ^2^ = 0.373. No difference emerged for self‐reported mental effort (blocked: *M* = −0.39, *SD* = 0.71; random: *M* = −0.20, *SD* = 0.96), *t*(30) = −0.63, *p* = 0.54, *r* ^2^ = 0.114.

#### 3.2.3 Alpha power

The Group × ROI × Time × Subset ANOVA on relative alpha power revealed main effects for ROI, *F*(5, 26) = 17.51, *p* < 0.001, λ = 0.229, $\eta_{p}^{2}$ = 0.564, and time, *F*(3, 28) = 6.38, *p* = 0.002, λ = 0.594, $\eta_{p}^{2}$ = 0.172. These effects were superseded by a ROI × Time interaction, *F*(15, 16) = 3.34, *p* = 0.01, λ = 0.242, $\eta_{p}^{2}$ = 0.285, revealing cubic temporal trends (i.e., increase, decrease, increase) for the frontal, *F*(1, 31) = 13.79, *p* = 0.001, $\eta_{p}^{2}$ = 0.308, left central, *F*(1, 31) = 13.75, *p* = 0.001, $\eta_{p}^{2}$ = 0.307, right central, *F*(1, 31) = 10.38, *p* = 0.003, η_p_^2^ = 0.251, and left temporal, *F*(1, 31) = 17.06, *p* < 0.001, $\eta_{p}^{2}$ = 0.355, regions. Changes in the occipital region were best described by a linear trend (i.e., decrease), *F*(1, 31) = 17.56, *p* < 0.001, eta = 0.362. No temporal trend emerged for the right temporal region. No main group effect was revealed, *F*(1, 30) = 1.59, *p* = 0.22, $\eta_{p}^{2}$ = 0.050. However, the ANOVA analysis also revealed interactions for ROI × Time × Subset, *F*(45, 1,350) = 2.03, *p* < 0.001, $\eta_{p}^{2}$ = 0.063, and Group × ROI × Time × Subset, *F*(45, 1,350) = 1.49, *p* = 0.02, $\eta_{p}^{2}$ = 0.047.

**Figure 3:**
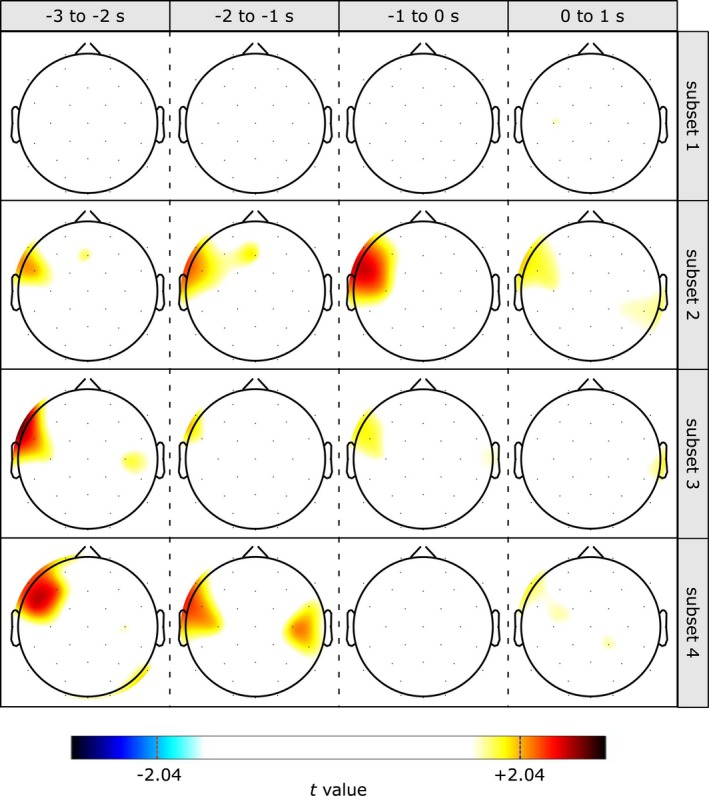
Scalp maps of independent samples *t* values comparing blocked versus random groups, as a function of time and subset. Values of −2.04 and 2.04 correspond to *p* = 0.05 on a *t* distribution with 30 *df* Positive values indicate greater whereas negative values indicate smaller relative alpha power for the blocked than the random group. Statistical thresholding was computed using the maximum‐statistic permutation testing (Cohen, 2014; Nichols & Holmes, 2002) controlling for multiple comparisons in the Channel × Time dimensions with alpha set at 0.01: no significant effect was revealed

Post hoc independent samples *t* tests revealed greater alpha power for the random compared to the blocked group for selected channels and only for Subsets 2 to 4 (Figure 3). For Subset 2, effects emerged from −3 to −2 s at FC5, *t*(30) = 2.07, *p* = 0.05, *r* ^2^ = 0.353, from −2 to −1 s at F7, *t*(30) = 2.08, *p* = 0.05, *r* ^2^ = 0.355, and FC5, *t*(30) = 2.04, *p* = 0.05, *r* ^2^ = 0.350, from −1 to 0 s at F7, *t*(30) = 2.29, *p* = 0.03, *r* ^2^ = 0.385, FC5, *t*(30) = 2.52, *p* = 0.02, *r* ^2^ = 0.418, and T7, *t*(30) = 2.09, *p* = 0.05, *r* ^2^ = 0.356. For Subset 3, effects emerged from −3 to −2 s at F7, *t*(30) = 2.62, *p* = 0.01, *r* ^2^ = 0.432, and FC5, *t*(30) = 2.27, *p* = 0.03, *r* ^2^ = 0.383. For Subset 4, effects emerged from −3 to −2 s at F7, *t*(30) = 2.31, *p* = 0.03, *r* ^2^ = 0.388, F3, *t*(30) = 2.27, *p* = 0.03, *r* ^2^ = 0.383, and FC5, *t*(30) = 2.48, *p* = 0.02, *r* ^2^ = 0.412, from −2 to −1 s at F7, *t*(30) = 2.14, *p* = 0.04, *r* ^2^ = 0.365, FC5, *t*(30) = 2.16, *p* = 0.04, *r* ^2^ = 0.367, T7, *t*(30) = 2.05, *p* = 0.05, *r* ^2^ = 0.350, and C4, *t*(30) = 2.06, *p* = 0.05, *r* ^2^ = 0.352. Permutation testing conducted to control for multiple comparisons in the Channel × Time dimensions indicated that the findings reported above did not survive a rigorous statistical control (Figure 3). The exploration of multiple frequency bands (theta, lower alpha, upper alpha, beta) revealed that the effects described above were not specific to the alpha frequency band, although they appeared especially distinctly for the upper alpha sub‐band (see Appendix S4).

### 3.3 Retention effects

To address our third study aim, we conducted analyses to explore the persistence of the effects due to the manipulation of target variability in an immediate retention test with no target variability.

#### 3.3.1 Performance

Performance at retention was better than baseline. However, no group differences emerged for radial error (blocked: *M* = −3.97, *SD* = 5.62 cm; random: *M* = −2.14, *SD* = 5.53 cm; *t*(30) = −0.93, *p* = 0.36, *r* ^2^ = 0.167), length error (blocked: *M* = −3.82, *SD* = 5.37 cm; random: *M* = −1.99, *SD* = 5.54 cm, *t*(30) = −0.95, *p* = 0.35, *r* ^2^ = 0.171), or angle error (blocked: *M* = −0.21, *SD* = 0.62 degrees; random: *M* = −0.11, *SD* = 0.40 degrees, *t*(30) = −0.57, *p* = 0.58, *r* ^2^ = 0.104).

#### 3.3.2 Self‐report

Compared to baseline, during the retention test self‐reported difficulty decreased similarly for the blocked (*M* = −0.88, *SD* = 1.20) and random (*M* = −0.0.56, *SD* = 1.41), groups,* t*(30) = −0.67, *p* = 0.51, *r* ^2^ = 0.121. Mental effort also decreased similarly from baseline to retention for the blocked (*M* = −0.75, *SD* = 0.68) and random (*M* = −0.88, *SD* = 0.96) groups, *t*(30) = 0.43, *p* = 0.67, *r* ^2^ = 0.078.

#### 3.3.3 Alpha power

The Group × ROI × Time ANOVA conducted on relative alpha power revealed main effects for ROI, *F*(5, 26) = 22.92, *p* < 0.001, λ = 0.185, $\eta_{p}^{2}$ = 0.530, and time, *F*(3, 28) = 5.39, *p* = 0.01, λ = 0.634, $\eta_{p}^{2}$ = 0.174. These main effects were superseded by a ROI × Time interaction, *F*(15, 16) = 3.93, *p* = 0.01, λ = 0.213, $\eta_{p}^{2}$ = 0.204, indicating that the time effect was best described by a cubic trend (i.e., increase, decrease, increase) for the frontal, *F*(1, 30) = 15.02, *p* = 0.001, $\eta_{p}^{2}$ = 0.334, right central, *F*(1, 30) = 4.25, *p* = 0.05, $\eta_{p}^{2}$ = 0.124, left temporal, *F*(1, 30) = 16.96, *p* < 0.001, $\eta_{p}^{2}$ = 361, and right temporal, *F*(1, 30) = 5.74, *p* = 0.02, $\eta_{p}^{2}$ = 0.161, regions. Changes in the occipital region were best described by a linear trend (i.e., decrease), *F*(1, 30) = 16.76, *p* < 0.001, $\eta_{p}^{2}$ = 0.358. No trend was evident for the left central region. No main effect for group emerged, *F*(1, 30) = 2.62, *p* = 0.12, $\eta_{p}^{2}$ = 0.080, or group interactions. Appendix S3 illustrates scalp maps along with the outcome of permutation testing conducted for group differences at retention. Appendix S4 reports the analyses conducted on multiple frequency bands (theta, lower alpha, upper alpha, beta).

## 4 DISCUSSION

This study investigated movement‐related alpha gating in a golf putting task. Our first goal was to establish the existence and behavioral relevance of a topographic pattern of alpha power compatible with the gating phenomenon in preparation for movement execution. Our second goal was to alter this gating phenomenon via an experimental manipulation of target variability by comparing blocked versus random schedules of movement repetitions. Our final goal was to evaluate the short‐term persistence of functional adaptations induced by the manipulation of target variability. The findings of this study are discussed below, separately with regard to each goal.

### 4.1 Alpha gating

Regional analyses of alpha power in the 2 s preceding movement execution revealed a focal pattern whereby alpha power was highest for the occipital region, intermediate for the bilateral temporal and frontal regions, and lowest for the bilateral central regions (Figure 2). Based on the proposed inhibitory function of cortical alpha (Klimesch, 2012; Klimesch et al., 2007) and the gating‐by‐inhibition model (Jensen & Mazaheri, 2010), our findings imply that cognitive activity was clearly diverted away from processes performed in the occipital region and, to a lesser extent, the temporal and frontal regions, and instead routed toward processes performed in the central regions. This finding provides further evidence of the existence of the aiming movement‐related alpha gating phenomenon (Gallicchio et al., 2017; Gallicchio, Finkenzeller, et al., 2016).

That the focal pattern of alpha power was specific to movement preparation is supported by analyses comparing the 2 s preceding movement initiation with the 2 s preceding cue onset (i.e., the stimulus prompting participants to start their putting preparation). These analyses revealed that, relative to the precue period, alpha power increased in the occipital and left temporal regions and decreased for the central and frontal regions (Figure 2c). No significant change occurred in the right temporal region. In other words, the gating pattern became more intense just before the start of movement execution. Our finding replicates that of Gallicchio, Finkenzeller, et al. (2016) who observed a focal topography of alpha power prior to rifle shooting but not at rest. These findings help consolidate the argument that the alpha gating phenomenon observed in motor tasks is specific to preparation for movement. The fact that a weaker alpha gating effect was evident in the precue periods (Figure 2a) may reflect a state of nascent preparation for movement in the time between one repetition and the next (cf. Cooke et al., 2015). The suppression of central alpha just before movement initiation is a well‐known phenomenon in the psychophysiological literature concerned with the study of movement, and it is interpreted as reflecting the activation of sensorimotor processes necessary for the execution of the movement (Neuper & Pfurtscheller, 2001; Pfurtscheller, 1992).

Finally, we observed that the intensity of the alpha gate was associated with the accuracy of the movement. Namely, participants with larger increases for occipital alpha performed better in terms of smaller radial and length errors (Figure 2d,g). Moreover, the association between performance and EEG power was evident only for the alpha frequency bands (see Appendix S4). This finding suggests that the inhibition of the occipital region is functionally implicated in the performance of target‐based motor tasks. It is worth noting that optimal programming of force and direction may rely on two distinct neural patterns because the topographies showing the correlation of alpha power with either length or angle errors appeared qualitatively different (Figure 2h,i).

### 4.2 Target variability

The most important purpose of the present study was to attempt to experimentally alter the alpha gating phenomenon by manipulating trial‐by‐trial target variability. At the behavioral level, individuals who putted to randomly varying targets within blocks reported greater task difficulty than those who putted to the same target within blocks. Accordingly, compared to baseline, performance during the scheduling manipulation declined for the random group (the ball finished 2.6 cm farther from the target) and improved for the blocked group (the ball finished 0.7 cm closer to the target). However, it should be noted that the self‐reported measure of mental effort was not different between the random group and blocked group.

Group differences in performance became particularly evident after participants had some time to adapt to the constant or random repetition schedule. Specifically, the random and blocked groups started to differ in the second subset of repetition, that is, after they had putted the first 20 putts in their respective condition: 20 putts to a random sequence of the four peripheral targets for the random group, and five consecutive putts to the same target for each of the four peripheral targets for the blocked group. All indices of performance (i.e., radial, length, and angle errors) indicated that the random group performed significantly worse than the blocked group, with large effect sizes. It is interesting to note that, for the blocked group, performance improved steadily (i.e., radial error kept decreasing) across all repetition subsets, whereas for the random group, performance decreased during the first two subsets and started to improve during the two final subsets (Table 1). The decline in movement accuracy when performing under the randomly varying conditions has been previously observed in golf putting tasks (e.g., Porter & Magill, 2010) and has been attributed to its greater cognitive load (Lee, et al., 1985; Shea & Morgan, 1979). Below, we consider how the neurophysiological data recorded in the present study can shed light on how the additional cognitive load might have interfered with the selective allocation of resources to relevant processes and inhibiting irrelevant processes.

Alpha power followed a temporal trend that can be best described as an initial increase, followed by a decrease (peaking at movement initiation), and a final increase (during movement execution). This pattern has been previously interpreted as the timely allocation of resources to motor preparation processes (Cooke et al., 2015). Importantly, the peak‐to‐trough pattern has been found to be greater for experts than novices (Cooke et al., 2014) and associated with larger performance improvements after training (Gallicchio et al., 2017).

Spatial analyses revealed that group differences were mostly localized to the left temporal region. More specifically, compared to the blocked group, the random group showed a reduced left temporal alpha activity across the 3 s preceding movement onset. Mirroring the results for performance, group differences emerged after the participants had time to adapt to the requirements of putting to either the same target or a randomly varying target (Figure 3). Because alpha reflects regional inhibition (Klimesch, 2012; Klimesch et al., 2007), this finding indicates that prior to movement initiation the left temporal region was inhibited less for the random group than the blocked group. Within the framework of the alpha gating‐by‐inhibition model (Jensen & Mazaheri, 2010), this result can be interpreted as deficient gating of cognitive resources across the cortex. In line with previous interpretations of the movement‐related alpha gating phenomenon (Gallicchio et al., 2017; Gallicchio, Finkenzeller, et al., 2016), the weaker alpha gate observed for the random group reflects less psychomotor efficiency (Hatfield, 2018; Hatfield & Hillman, 2001) compared to the blocked group. These novel findings provide evidence that increased inhibition of cortical regions that are not involved with movement seems to be more important than increased activation of regions that are responsible for movement control. Indeed, Gallicchio et al. (2017) found that larger improvements in putting performance were associated with stronger inhibition of nonmotor regions rather than greater activation of motor regions.

It is worth pointing out that the left temporal region appears to play a special role in the control and learning of motor skills. Previous investigations have observed greater alpha power (i.e., stronger inhibition) in the left temporal region as a function of expertise (Haufler, Spalding, Santa Maria, & Hatfield, 2000), performance (Gallicchio, Finkenzeller, et al., 2016), and training (Gallicchio et al., 2017; Kerick, Douglass, & Hatfield, 2004; Landers et al., 1994). Past studies have also found increased functional disconnection between the left temporal region and other regions involved with movement as a function of expertise (Deeny, Hillman, Janelle, & Hatfield, 2003), performance (Gallicchio, Cooke, & Ring, 2016), and training (Gallicchio et al., 2017; Zhu, Poolton, Wilson, Maxwell, & Masters, 2011). Left temporal activity in a movement task has been interpreted as cognitive/verbal interference during motor preparation (Deeny et al., 2003) and reinvestment of declarative knowledge to consciously monitor and control movements (Zhu et al., 2011). It is tempting to speculate that verbal processes concerned with use of declarative knowledge may impair motor performance and hold back progress in learning of a motor skill. However, due to the unfeasibility of mapping one region to one cognitive function, we urge researchers to study the function of left temporal alpha in motor tasks.

### 4.3 Retention effects

The evaluation of group differences at retention enabled us to test predictions derived from motor learning theories that have argued for a learning advantage following variable practice (Schmidt, 1975). Contrary to our expectations, no behavioral or neurophysiological differences emerged at retention, when both groups putted repeatedly to the same central target. The lack of effects could be attributed to the short duration of the putting practice: participants may have not accumulated sufficient practice during one session of only 80 repetitions. The lack of effects may also be attributed to the fact that the post‐test assessment was a retention rather than a transfer test (i.e., participants putted to the same target as baseline rather than to a different target). It is also likely that the participants had a certain degree of neural efficiency at baseline (Figure 2): the random variability practice posed a temporary challenge to this efficiency, but then functional activity reverted to its initial pattern once the challenge had been removed. These issues can be addressed in future studies.

### 4.4 Limitations and directions for future research

The findings of the present study shed light on the neurophysiological mechanisms underlying variable practice. However, in order to appreciate the applicability of our findings and identify directions for future research, we need to acknowledge some study limitations. First, the fact that, compared to the blocked group, the random group performed worse (i.e., made larger errors) may have contributed to group differences in alpha gating. Namely, the production of larger errors may have resulted in enhanced cognitive activity aimed at improving performance on the next trial (Cooke et al., 2015).

Second, this study failed to find any retention effects after practicing under variable conditions. Future research could adopt a longitudinal design to explore the impact of longer trainings that could result in a more stable functional adaptation. It would be interesting to examine whether repetition under a variable schedule leads eventually to performance improvements and to identify the neurophysiological mechanisms explaining such an improvement.

Third, most findings of this study are in regard to activity detected by sensors located at the edge of the spatial configuration examined. Due to our efforts in attenuating movement artifacts, we have interpreted these effects in terms of cortical activity. However, we cannot rule out that non‐neural activity, such as neck movements, has influenced our results. We recommend that future research pay particular attention to this issue. For instance, researchers could record head movements through accelerometers and identify the independent components that are statistically related to this signal (cf. Daly, Billinger, Scherer, & Müller‐Putz, 2013).

Finally, due to the compelling body of literature implicating the left temporal region in motor control and learning (Deeny et al., 2003; Gallicchio, Cooke, et al., 2016, 2017; Gallicchio, Finkenzeller, et al., 2016; Haufler et al., 2000; Kerick et al., 2004; Landers et al., 1994; Zhu et al., 2011), we recommend that researchers address the pressing issue of explaining the functional role being played by this region in movement tasks. For example, if inhibition of the left temporal region is linked to decreased verbal activity (e.g., Zhu et al., 2011), then future studies could manipulate self‐talk and look for associated changes in neurophysiology.

### 4.5 Conclusions

The present study demonstrated the explanatory utility of the alpha gating model in motor control research. We provided evidence of the existence of movement‐related alpha gating and its relevance for motor performance. We also demonstrated that this phenomenon can be changed in an experimental setting by manipulating variability across repetitions of the movement. The utility of a theoretical model, such as the alpha gating model, accounting for improvement in motor performance opens interesting avenues for future applications to enhance motor acquisition and evaluate the quality of training programs.

Our findings indicate that inhibition of occipital and temporal regions enhances performance in a target‐based motor task. Because these regions are involved with cognitive processes, including visual perception and retrieval of declarative knowledge, we could speculate that, once the spatial features of the target and movement parameters, such as force and direction, have been internalized as a mental representation of an action plan, further rumination in terms of visual processing and declarative thoughts may hinder rather than support performance. Accordingly, our evidence‐based recommendation to athletes at this stage would be: “Don't look, don't think, just do it!”.
