# Supplementary material for: Don’t look, don’t think, just do it! Toward an understanding of alpha gating in a discrete aiming task
Source: Psychophysiology. 2018 Oct 25;56(3):e13298. doi: 10.1111/psyp.13298 (PMC6849619; doi:10.1111/psyp.13298)
Supplement: Supplementary file 1 [file PSYP-56-na-s001.pdf]

Psychophysiology

Don't look, don't think, just do it! Towards an understanding of alpha gating in a discrete aiming task.

Germano Gallicchio and Christopher Ring

School of Sport, Exercise & Rehabilitation Sciences, University of Birmingham, Birmingham, United Kingdom

Appendix 1: Performance scoring

Photos of the putting surface were taken after each putt through a camera positioned on the ceiling (**Figure A1.1**) and were then imported into MATLAB for scoring. The scoring procedure consisted of three parts. First, a programme identified the coordinates of the targets and estimated the coordinates of the ball position prior to putting (**Figure A1.2**). Second, the coordinates of the final position of the ball were identified for each putt (**Figure A1.3**).

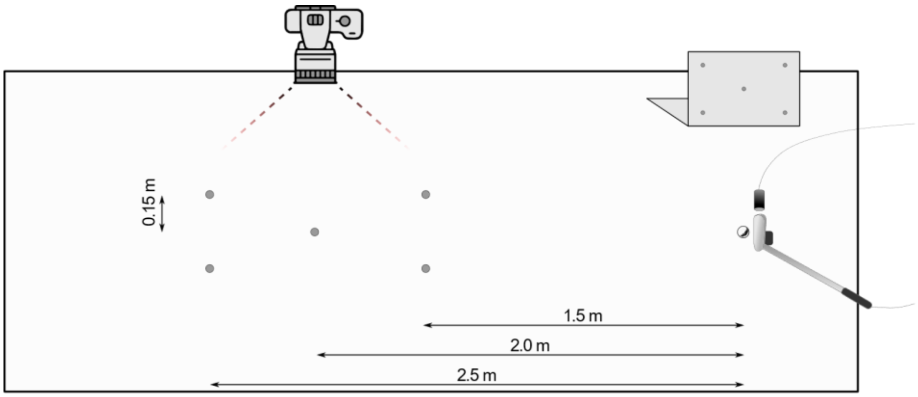

FIGURE A1.1 Putting task setup.

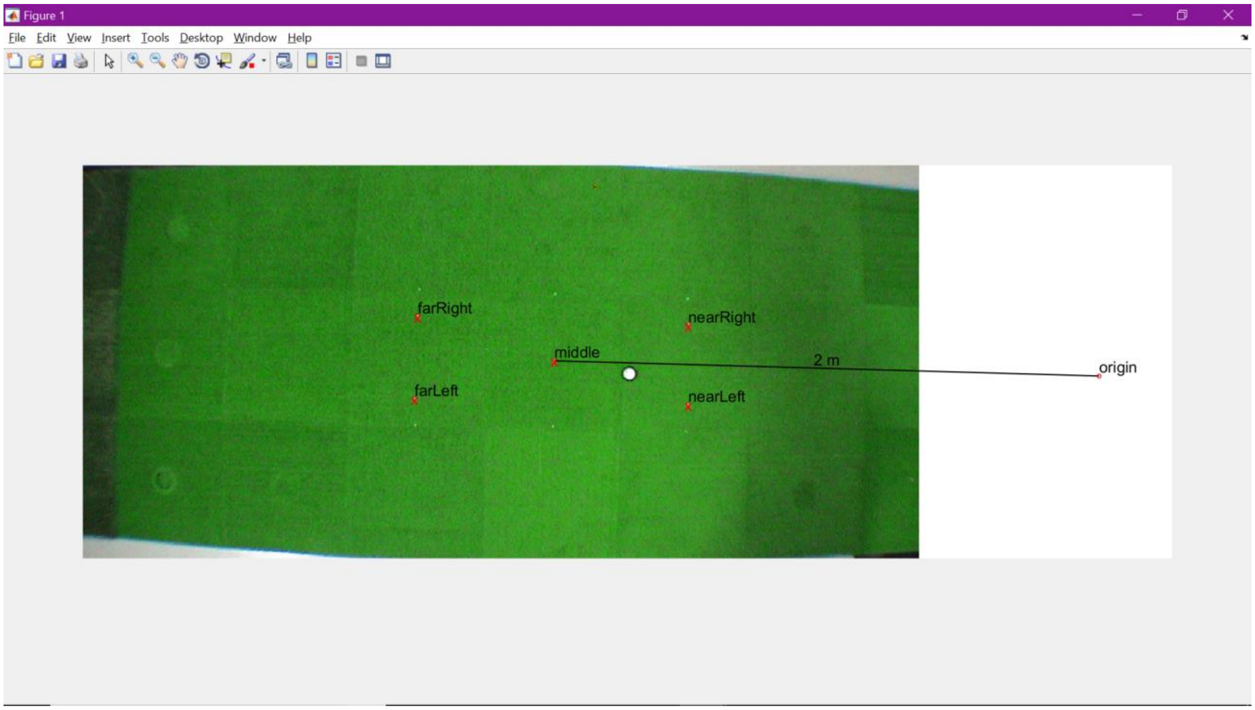

FIGURE A1.2 Identification of target coordinates (“middle”, “farLeft”, “farRight”, “nearLeft”, and “nearRight”) and starting position (“origin”; outside visual field of the camera).

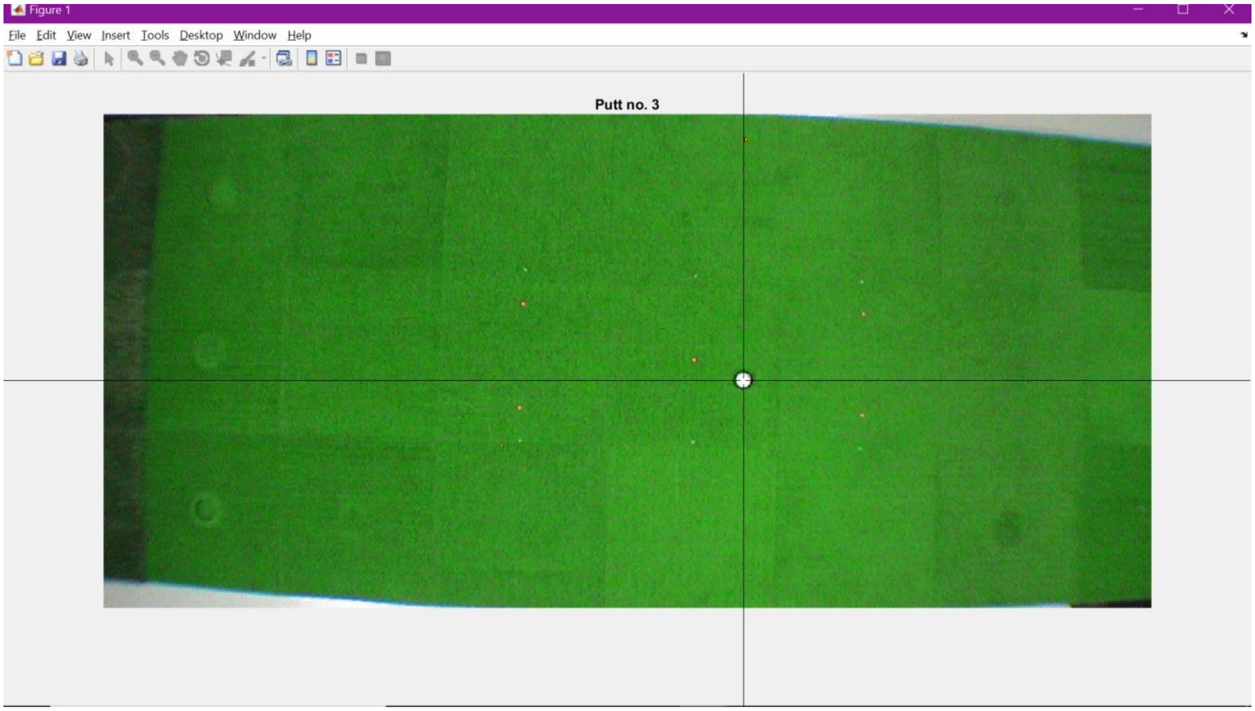

FIGURE A1.3 Identification of the coordinates of the final position of the ball.

Finally, target and ball coordinates were used to compute the following metrics: (1) target distance, as the distance of the target from the starting position; (2) target angle, as the angle of the target from the line connecting the middle target to the starting position; (3) ball distance, as the distance travelled by the ball from the starting position; (4) ball angle, as the angle of the final position of the ball from the line connecting the middle target to the starting position; (5) radial error, as the distance of the final position of the ball from the target (**Figure A1.4**); (6) length error, as the difference between the distance travelled by the ball from the starting position and the distance of the target from the starting position; (7) length absolute error, as the absolute value of the length error; (8) angle error, as the difference between the angle of the target from the line connecting the middle target to the starting position and the angle of the final position of the ball from the line connecting the middle target to the starting position; (9) angle absolute error, as the absolute value of the angle error.

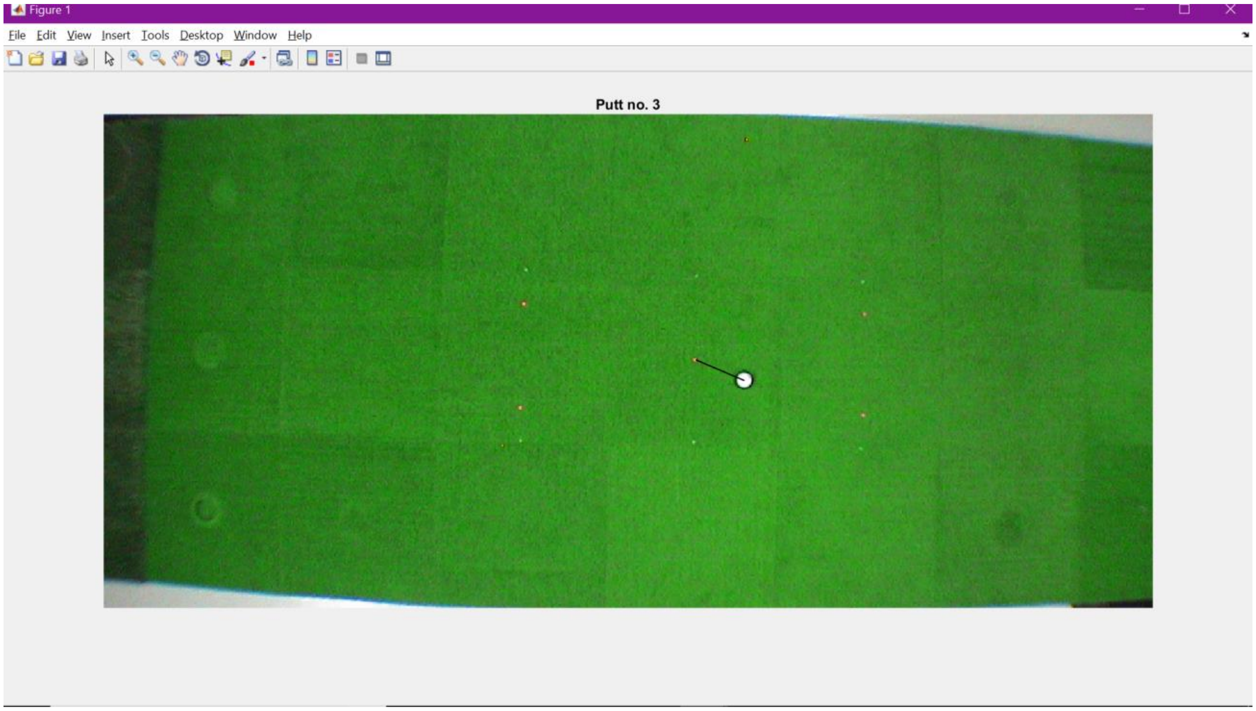

FIGURE A1.4 Radial error was computed as the distance from target and the final position of the ball.
