# Supplementary material for: Don’t look, don’t think, just do it! Toward an understanding of alpha gating in a discrete aiming task
Source: Psychophysiology. 2018 Oct 25;56(3):e13298. doi: 10.1111/psyp.13298 (PMC6849619; doi:10.1111/psyp.13298)
Supplement: Supplementary file 2 [file PSYP-56-na-s002.pdf]

Psychophysiology

Don’t look, don’t think, just do it! Towards an understanding of alpha gating in a discrete aiming task.

Germano Gallicchio and Christopher Ring

School of Sport, Exercise & Rehabilitation Sciences, University of Birmingham, Birmingham, United Kingdom

Appendix 2: Additional analyses of performance

In addition to evaluating performance as change scores from the baseline condition (see main text), we analysed group differences for performance separately for each condition (**Table A2.1**). Furthermore, we evaluated group differences for each target used in the test condition (**Table A2.2**).

Table A2.1 Mean (*SD*) of performance measures (i.e., radial, length, and angle error) in each condition (baseline, test, retention) separately per group (blocked, random) along with the results of the independent-sample t-tests.

Performance measure	Blocked	Random	$t(30)$	p	r^2
Baseline					
radial error (cm)	23.93 (4.74)	21.35 (5.99)	1.35	.19	.239
length error (cm)	22.61 (4.45)	20.44 (5.94)	1.17	.25	.209
angle error (degrees)	1.35 (0.55)	1.04 (0.38)	1.82	.08	.315
Test					
radial error (cm)	21.34 (3.98)	22.01 (3.59)	-0.50	.62	.091
length error (cm)	19.59 (3.99)	20.21 (3.82)	-0.45	.66	.082
angle error (degrees)	1.57 (0.33)	1.57 (0.22)	0.01	.99	.002
Retention					
radial error (cm)	19.96 (4.94)	19.22 (4.13)	0.46	.65	.084
length error (cm)	18.80 (4.89)	18.46 (4.16)	0.21	.83	.038
angle error (degrees)	1.14 (0.37)	0.94 (0.31)	1.66	.11	.290

Table A2.2 Mean (*SD*) of performance measures (i.e., radial, length, and angle error) for each target used in the test condition (i.e., far left, far right, near left, near right) separately per group (blocked, random) along with the results of the independent-sample t-tests.

Performance measure	Blocked	Random	$t(30)$	p	r^2
far left					
radial error (cm)	26.16 (6.69)	28.83 (5.18)	-1.26	.22	.224
length absolute error (cm)	23.89 (6.95)	26.1 (5.94)	-0.96	.34	.173
angle absolute error (degrees)	1.63 (0.35)	1.77 (0.46)	-0.94	.35	.169
far right					
radial error (cm)	26.56 (6.49)	25.83 (4.58)	0.37	.72	.067
length absolute error (cm)	24.50 (6.28)	23.71 (4.56)	0.41	.69	.075
angle absolute error (degrees)	1.52 (0.46)	1.53 (0.46)	-0.02	.98	.004
near left					
radial error (cm)	17.09 (4.05)	16.02 (3.85)	0.77	.45	.139
length absolute error (cm)	15.84 (4.06)	14.72 (3.97)	0.79	.43	.143
angle absolute error (degrees)	1.44 (0.71)	1.44 (0.61)	-0.02	.99	.004
near right					
radial error (cm)	15.35 (2.93)	17.36 (3.96)	-1.64	.11	.287
length absolute error (cm)	13.95 (3.06)	16.31 (3.99)	-1.87	.07	.323
angle absolute error (degrees)	1.66 (0.48)	1.53 (0.43)	0.84	.41	.152
