# Supplementary material for: Don’t look, don’t think, just do it! Toward an understanding of alpha gating in a discrete aiming task
Source: Psychophysiology. 2018 Oct 25;56(3):e13298. doi: 10.1111/psyp.13298 (PMC6849619; doi:10.1111/psyp.13298)
Supplement: Supplementary file 3 [file PSYP-56-na-s003.pdf]

Psychophysiology

Don’t look, don’t think, just do it! Towards an understanding of alpha gating in a discrete aiming task.

Germano Gallicchio and Christopher Ring

School of Sport, Exercise & Rehabilitation Sciences, University of Birmingham, Birmingham, United Kingdom

Appendix 3: Additional analyses of alpha power

Group differences at baseline. In order to assess group differences prior to manipulation of target variability, we conducted a 2 group × 6 ROI × 4 time mixed ANOVA on relative alpha power on the trials of the baseline condition. This analysis yielded a main effect for ROI, $F(5,26) = 20.60$, $p < .001$, $\lambda = .202$, $\eta_p^2 = .569$, indicating that relative alpha power was lowest for the frontal, left central, and right central regions, higher for left and right temporal regions, and highest for the occipital region. This regional pattern is consistent with the alpha gating phenomenon (see main text). No group effects or interactions were revealed (group: $F(1,30) = 0.57$, $p = .46$, $\eta_p^2 = .019$; group × ROI: $F(5,150) = 0.13$, $p = .99$, $\eta_p^2 = .004$; group × time: $F(3,90) = 1.45$, $p = .23$, $\eta_p^2 = .046$; group × ROI × time: $F(15,450) = 0.67$, $p = .82$, $\eta_p^2 = .022$). **Figure A3.1** shows the topography of independent-sample *t* values conducted to examine group differences at baseline (top panel), test (middle panel), and retention (bottom panel).

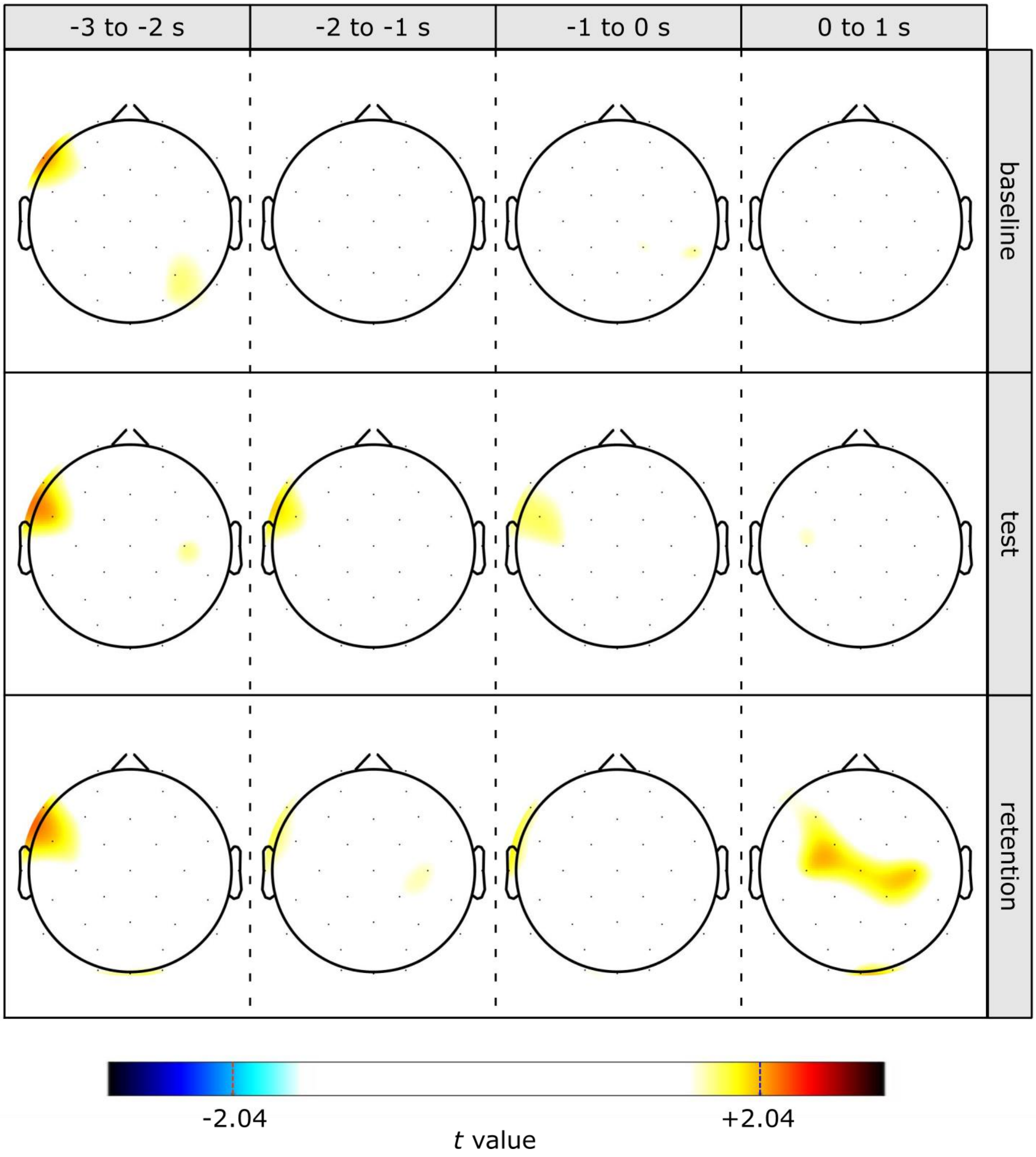

FIGURE A3.1 Scalp maps of independent-sample *t* values comparing blocked versus random groups as a function of time and condition (i.e., baseline, test, retention). Baseline and retention conditions included 20 trials each, whereas the test condition included 80 trials. Values of -2.04 and 2.04 correspond to p = .05 on a t distribution with 30 *df*. Positive values indicate greater whereas negative values indicate smaller relative alpha power for the blocked than the random group. Statistical thresholding was computed using the maximum-statistic permutation testing (Cohen, 2014; Nichols & Holmes, 2001) controlling for multiple comparisons in the channel × time dimensions with alpha set at .01: no significant effect was revealed.
