# Supplementary material for: Don’t look, don’t think, just do it! Toward an understanding of alpha gating in a discrete aiming task
Source: Psychophysiology. 2018 Oct 25;56(3):e13298. doi: 10.1111/psyp.13298 (PMC6849619; doi:10.1111/psyp.13298)
Supplement: Supplementary file 4 [file PSYP-56-na-s004.pdf]

Psychophysiology

Don’t look, don’t think, just do it! Towards an understanding of alpha gating in a discrete aiming task.

Germano Gallicchio and Christopher Ring

School of Sport, Exercise & Rehabilitation Sciences, University of Birmingham, Birmingham, United Kingdom

Appendix 4: Exploratory analyses of power in multiple frequency bands

We conducted additional analyses of EEG power in multiple frequency bands: theta (4-6 Hz), lower alpha (8-10 Hz), upper alpha (10-12 Hz), and beta (15-25 Hz). Figures A4.1, A4.2, A4.3, and A4.4 are equivalent to Figure 1 (main text); Figures A4.5, A4.6, A4.7, and A4.8 are equivalent to Figure 2 (main text); Figures A4.9, A4.10, A4.11, and A4.12 are equivalent to Figure A3.1 (Appendix 3).

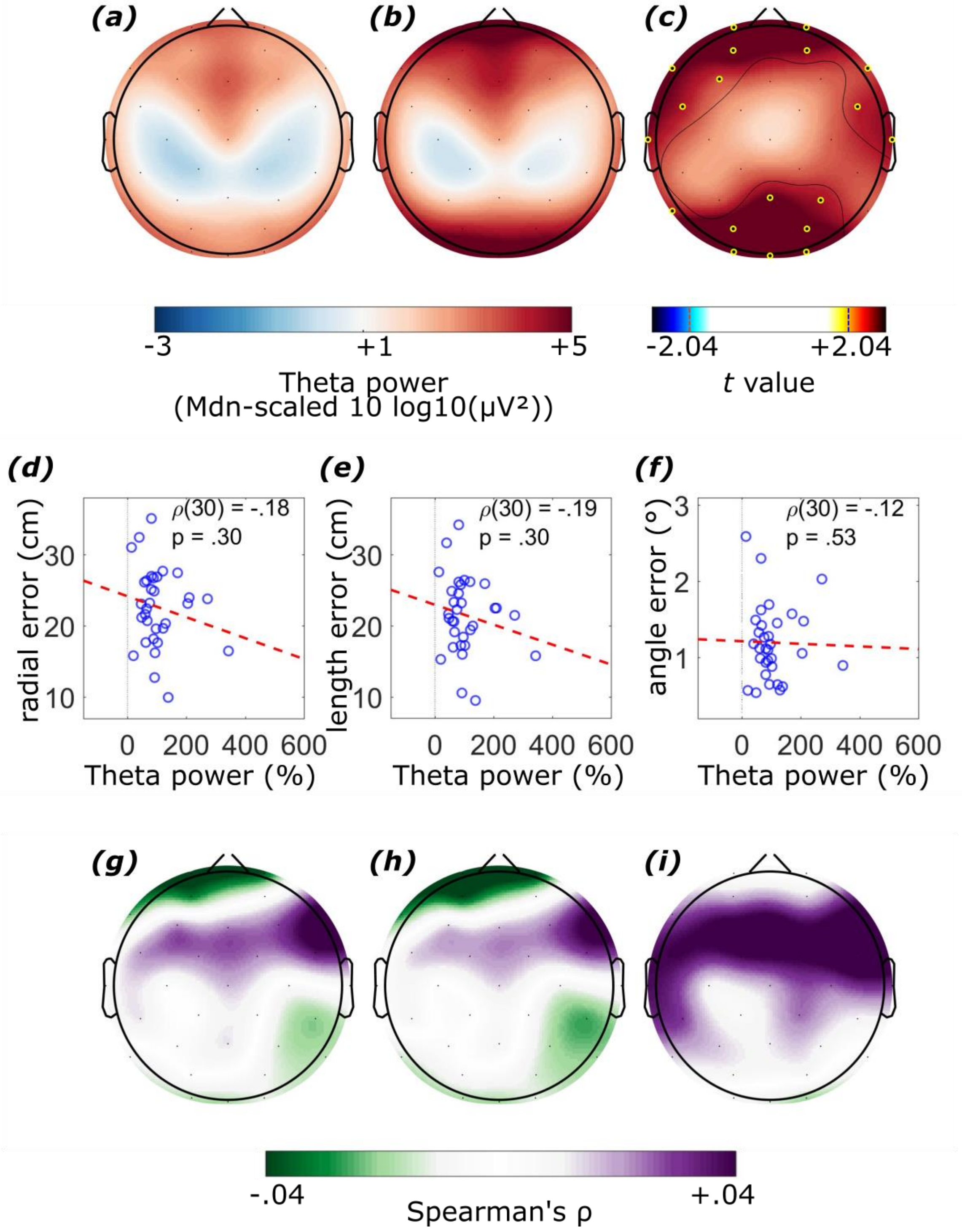

FIGURE A4.1 Replicate of Figure 1 (main text) for the theta band (4-6 Hz).

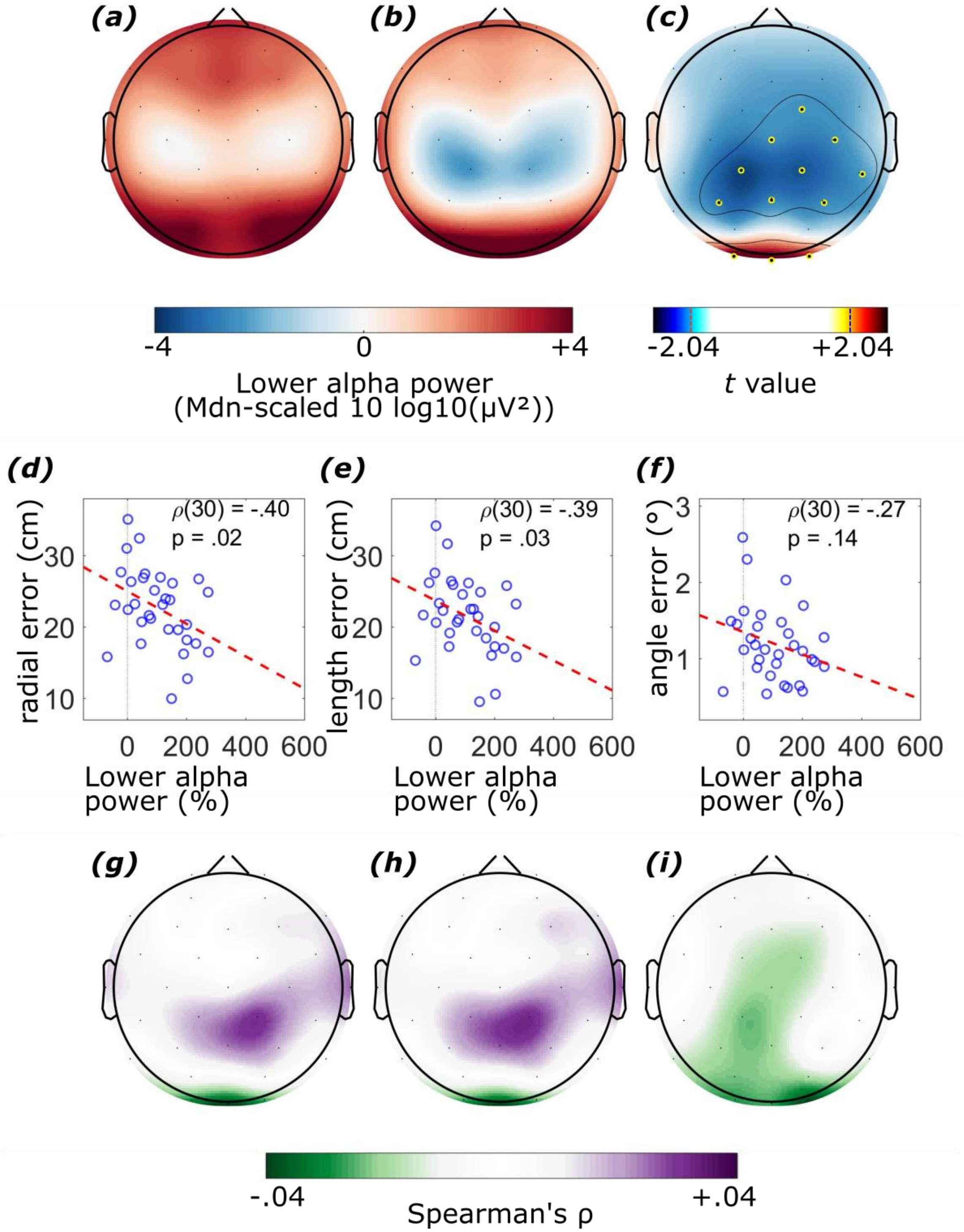

FIGURE A4.2 Replicate of Figure 1 (main text) for the lower alpha band (8-10 Hz).

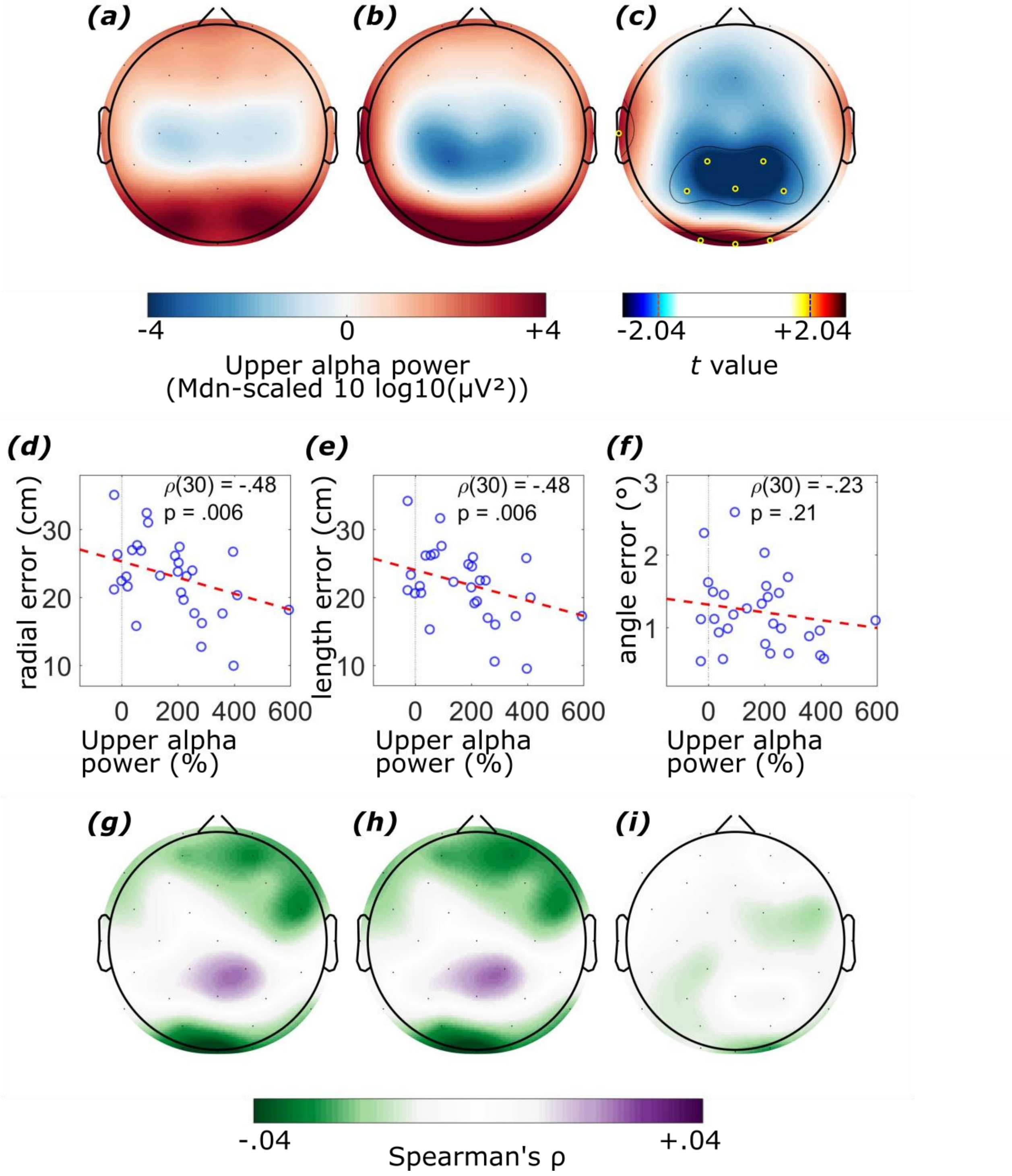

FIGURE A4.3 Replicate of Figure 1 (main text) for the upper alpha band (10-12 Hz).

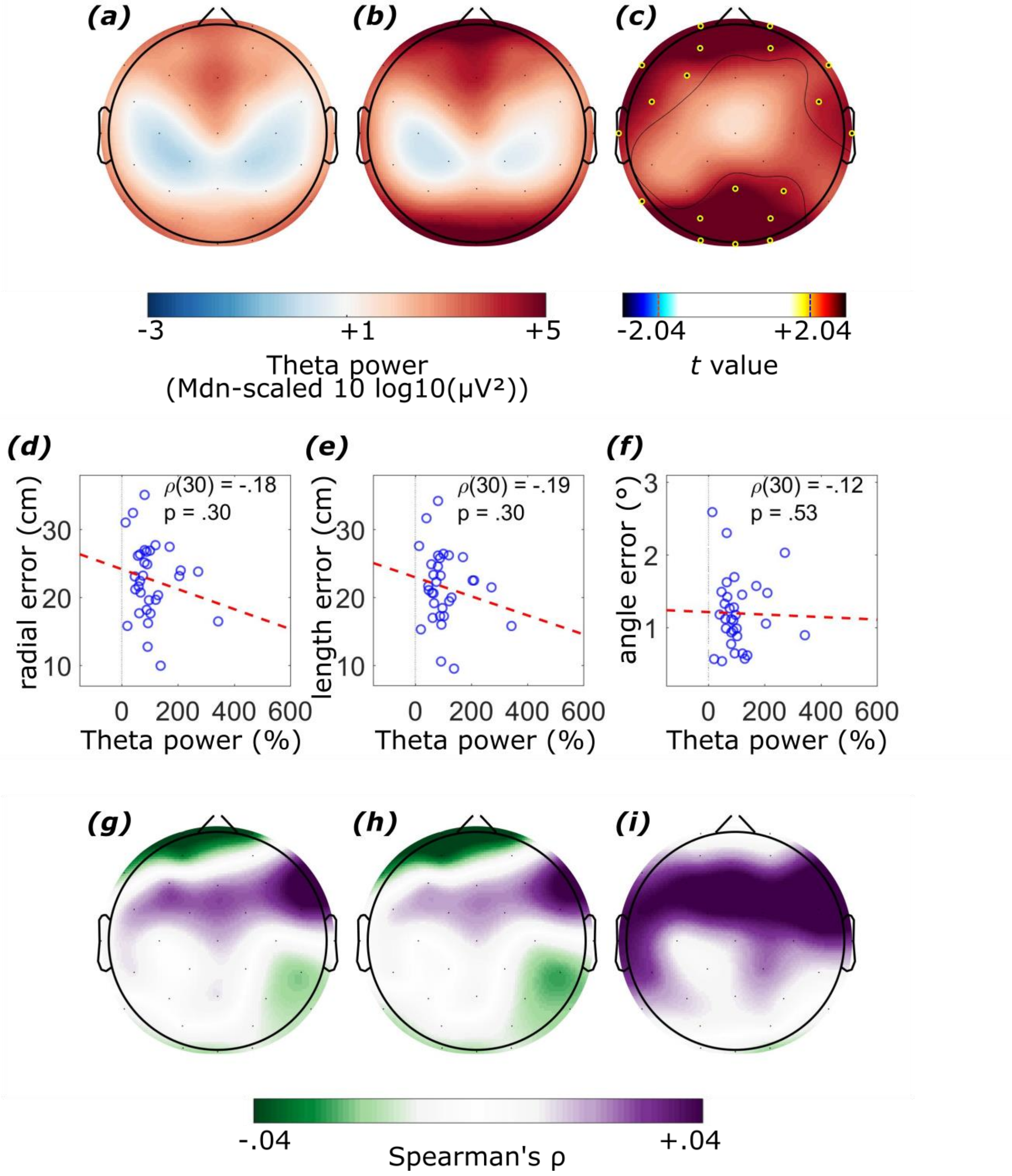

FIGURE A4.4 Replicate of Figure 1 (main text) for the beta band (15-25 Hz).

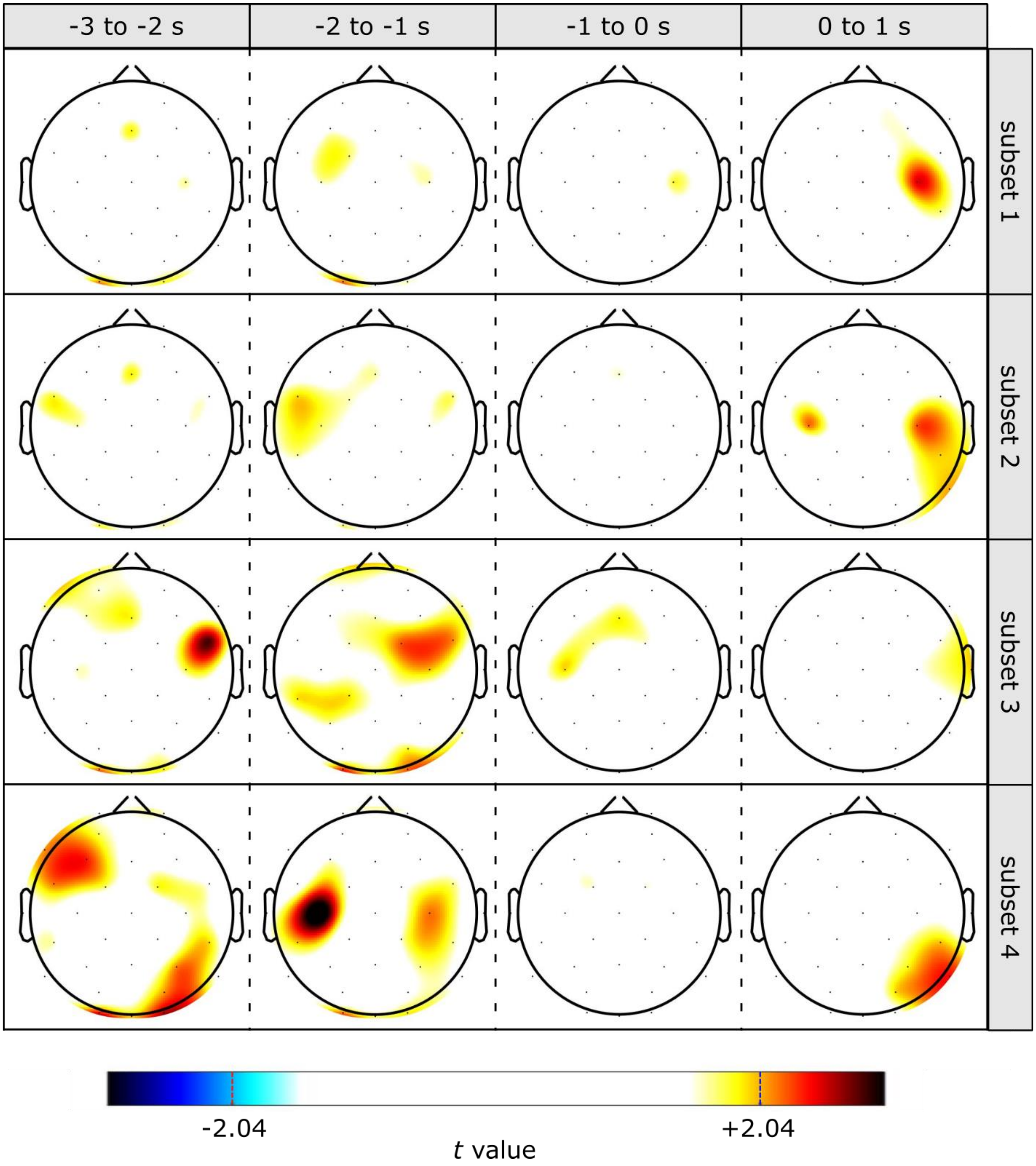

FIGURE A4.5 Replicate of Figure 2 (main text) for the theta band (4-6 Hz).

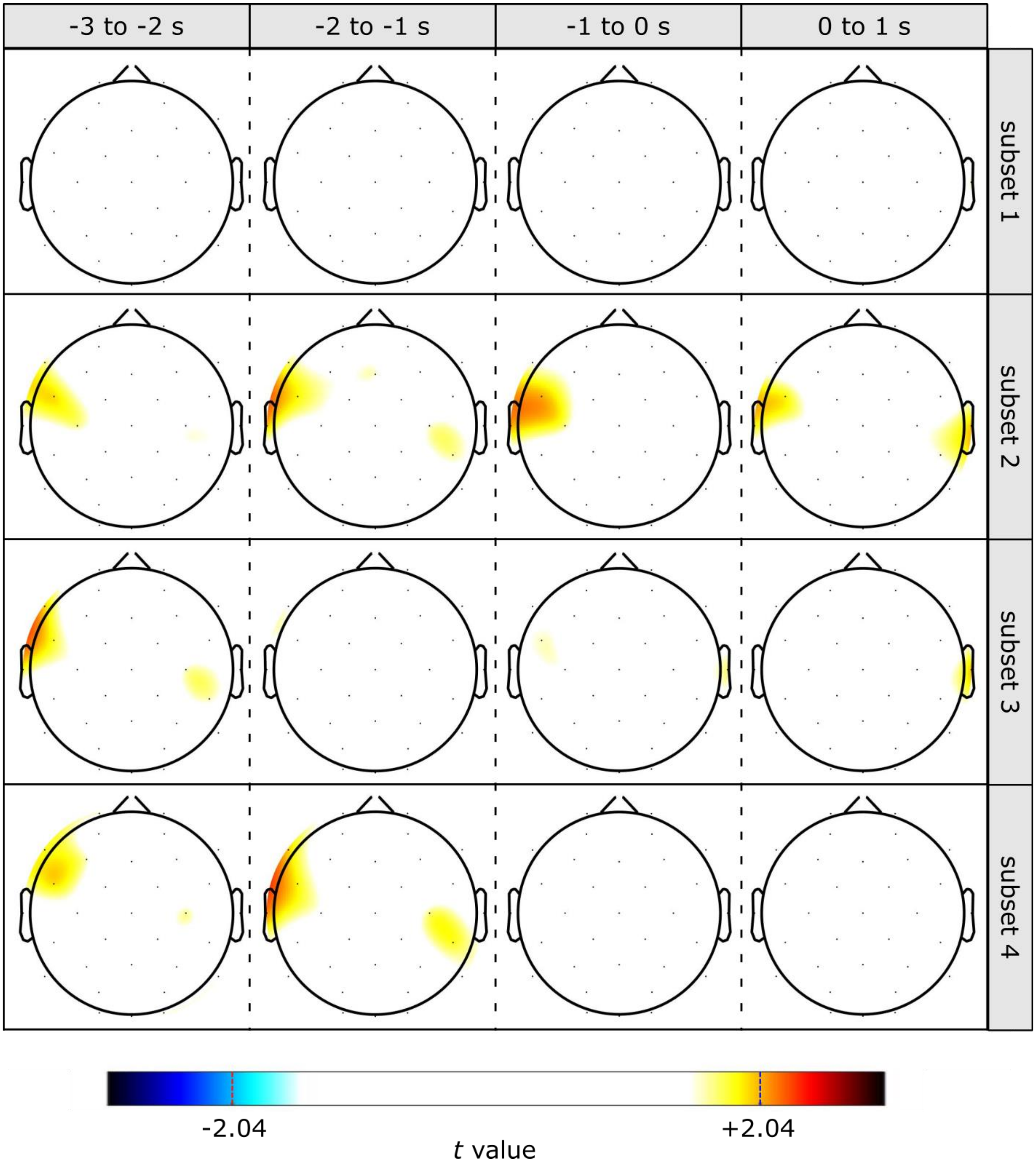

FIGURE A4.6 Replicate of Figure 2 (main text) for the lower alpha band (8-10 Hz).

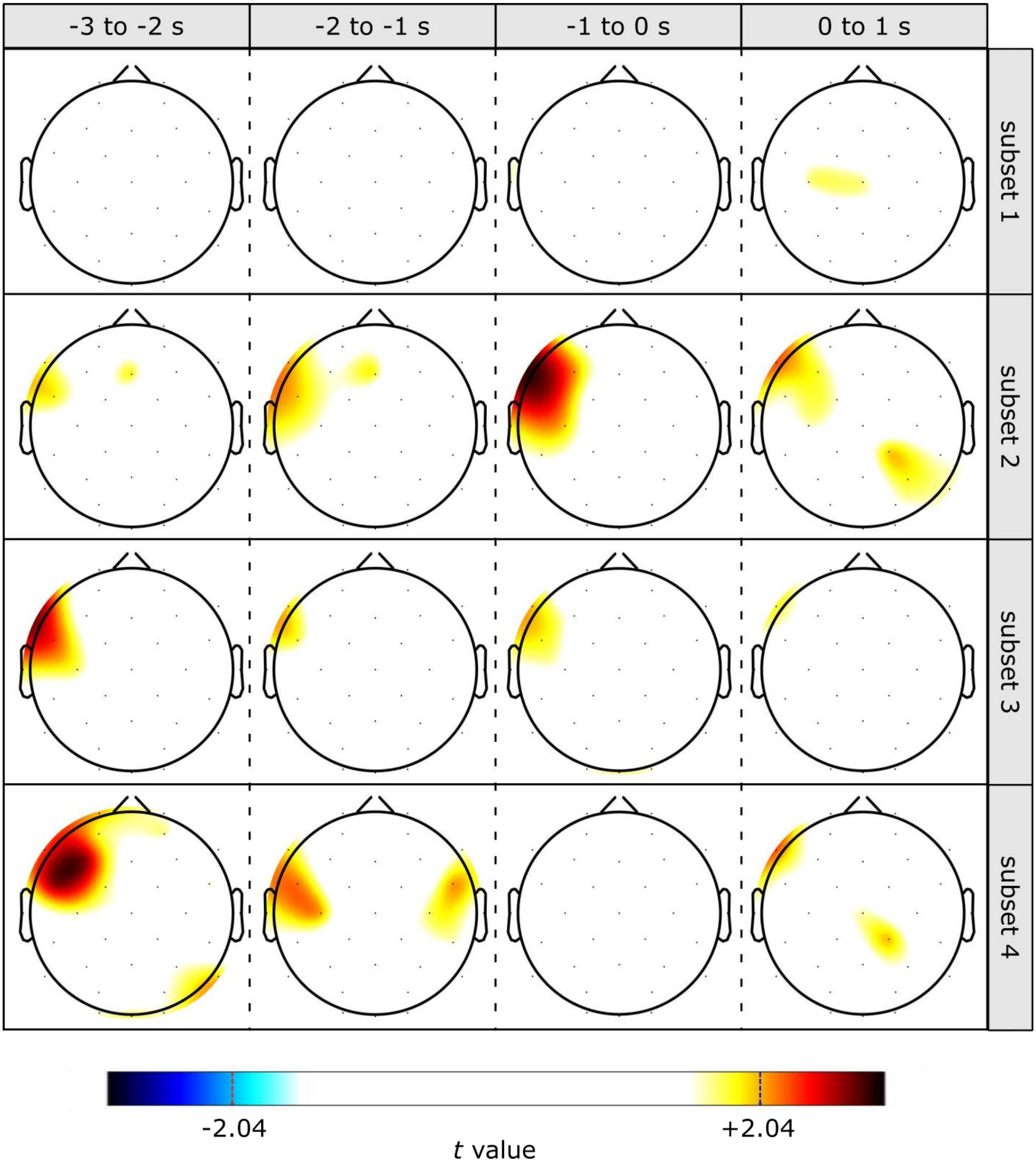

FIGURE A4.7 Replicate of Figure 2 (main text) for the upper alpha band (10-12 Hz).

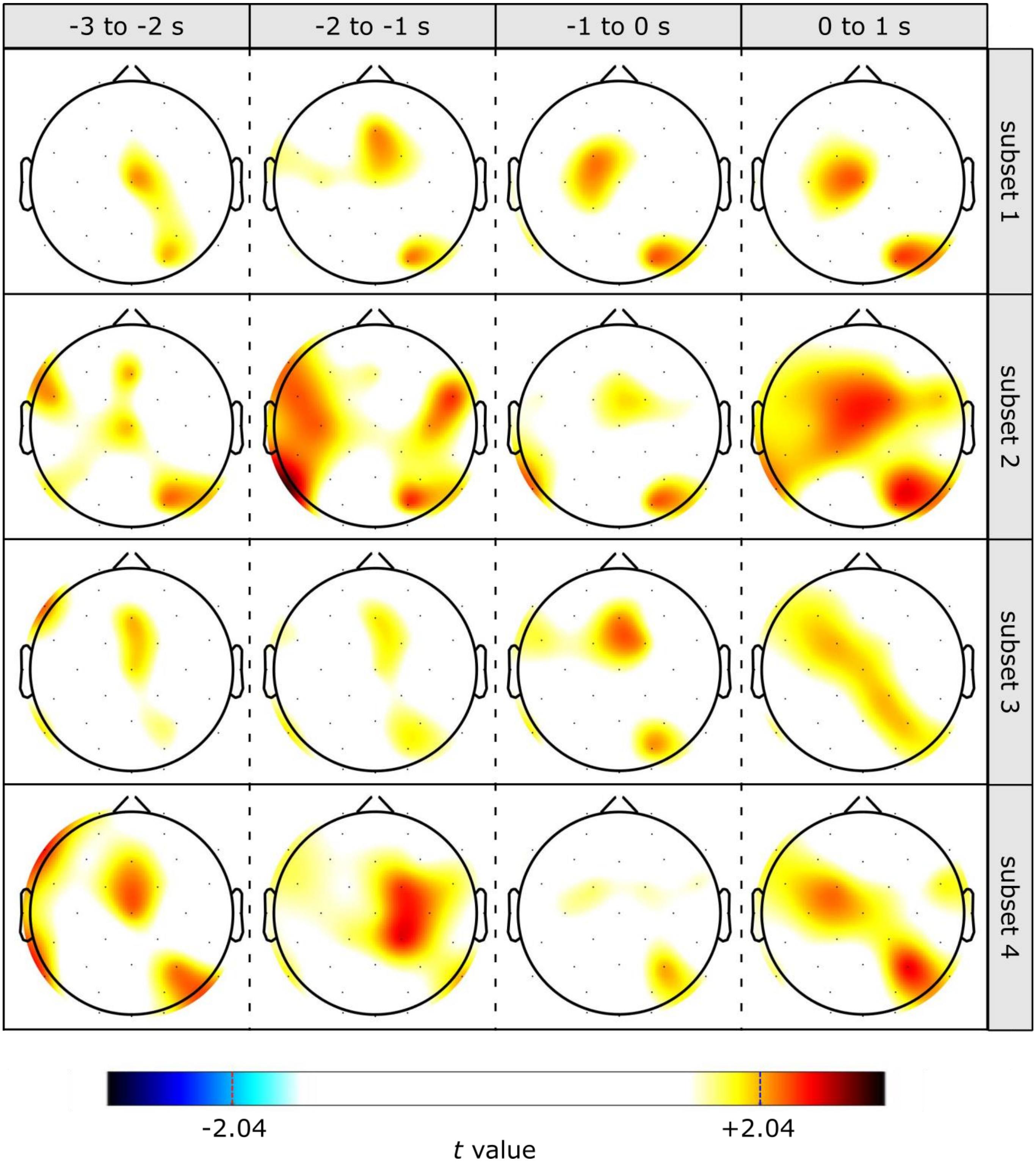

FIGURE A4.8 Replicate of Figure 2 (main text) for the beta band (15-25 Hz).

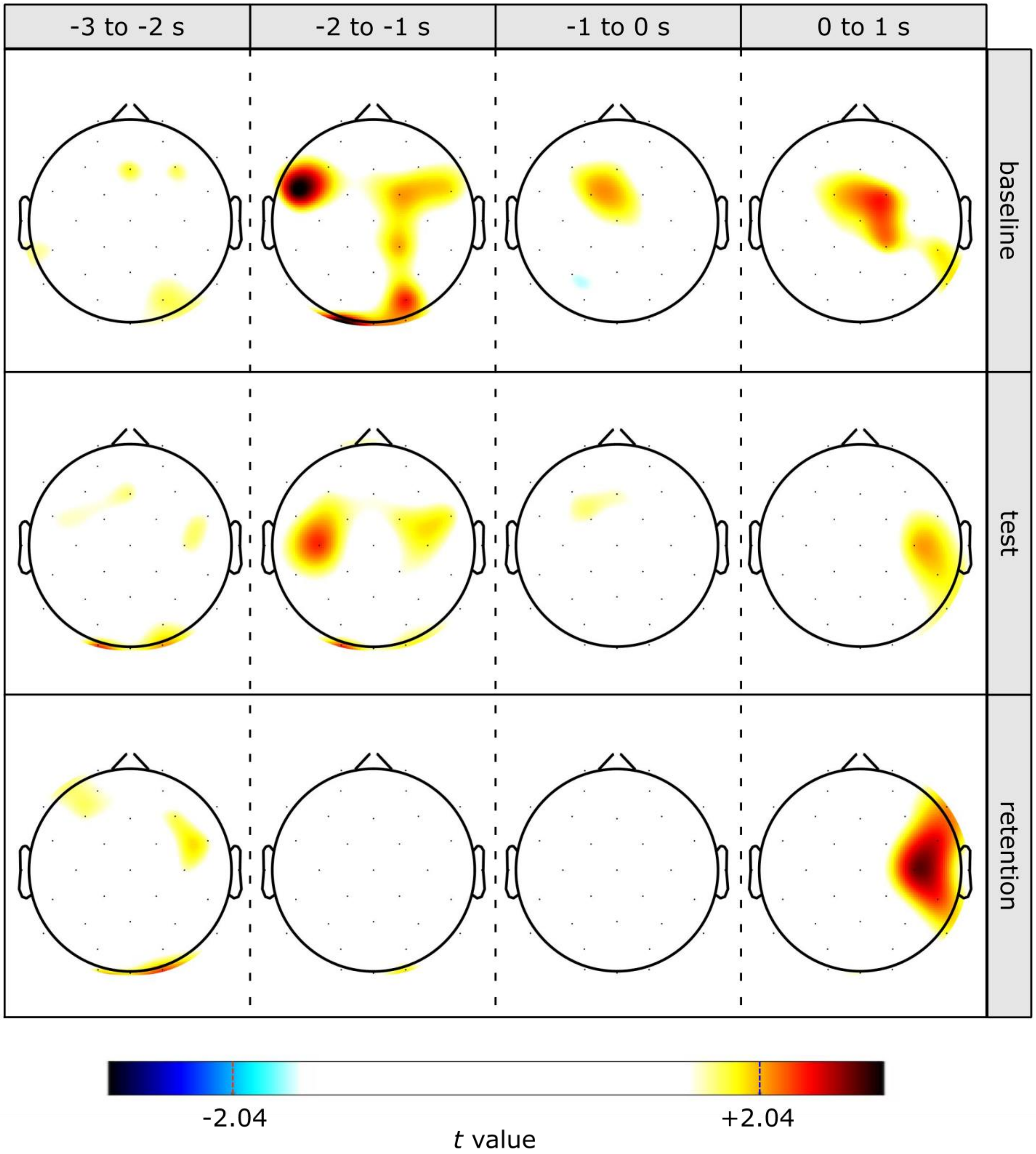

FIGURE A4.9 Replicate of Figure A3.1 (Appendix 3) for the theta band (4-6 Hz).

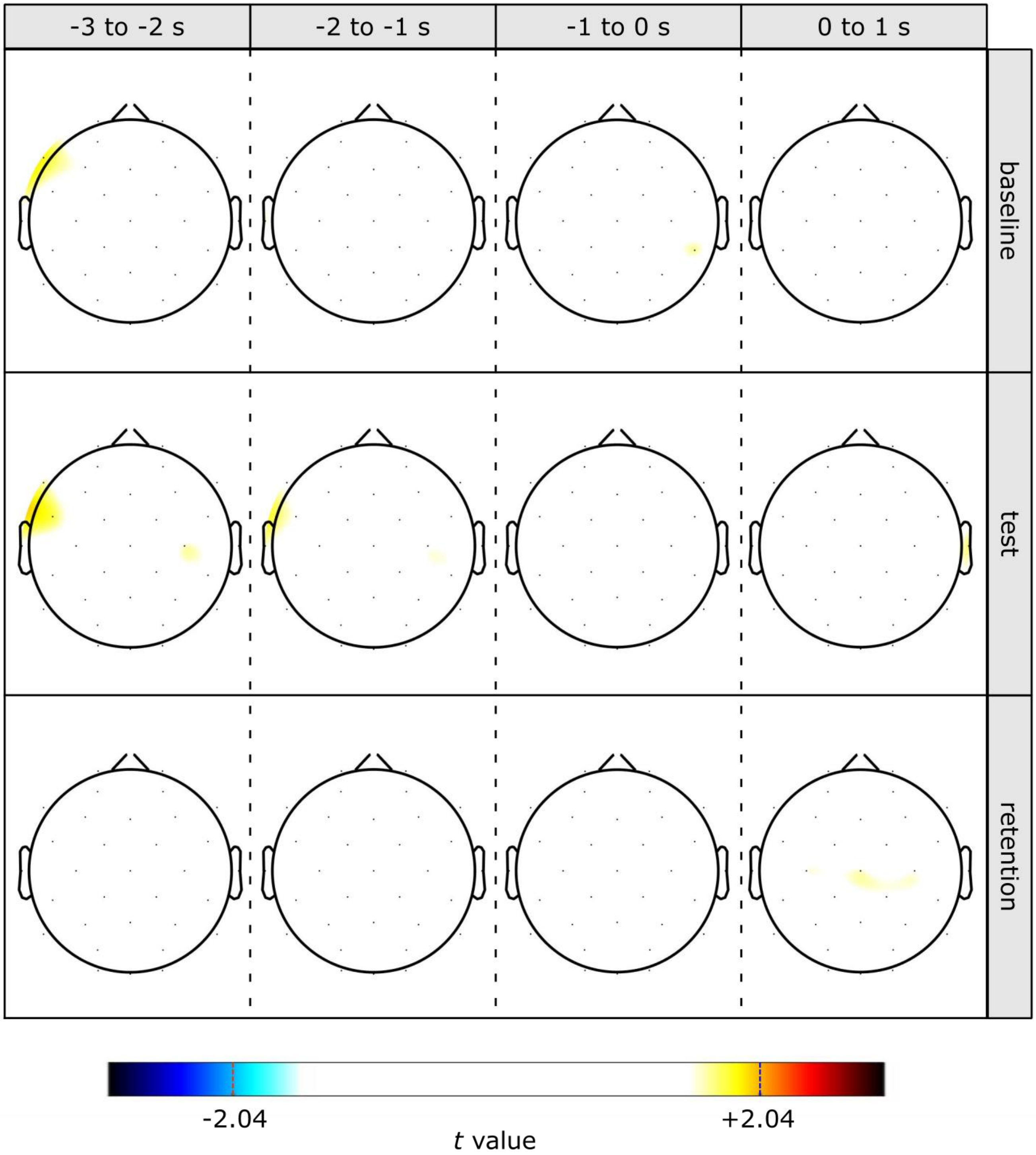

FIGURE A4.10 Replicate of Figure A3.1 (Appendix 3) for the lower alpha band (8-10 Hz).

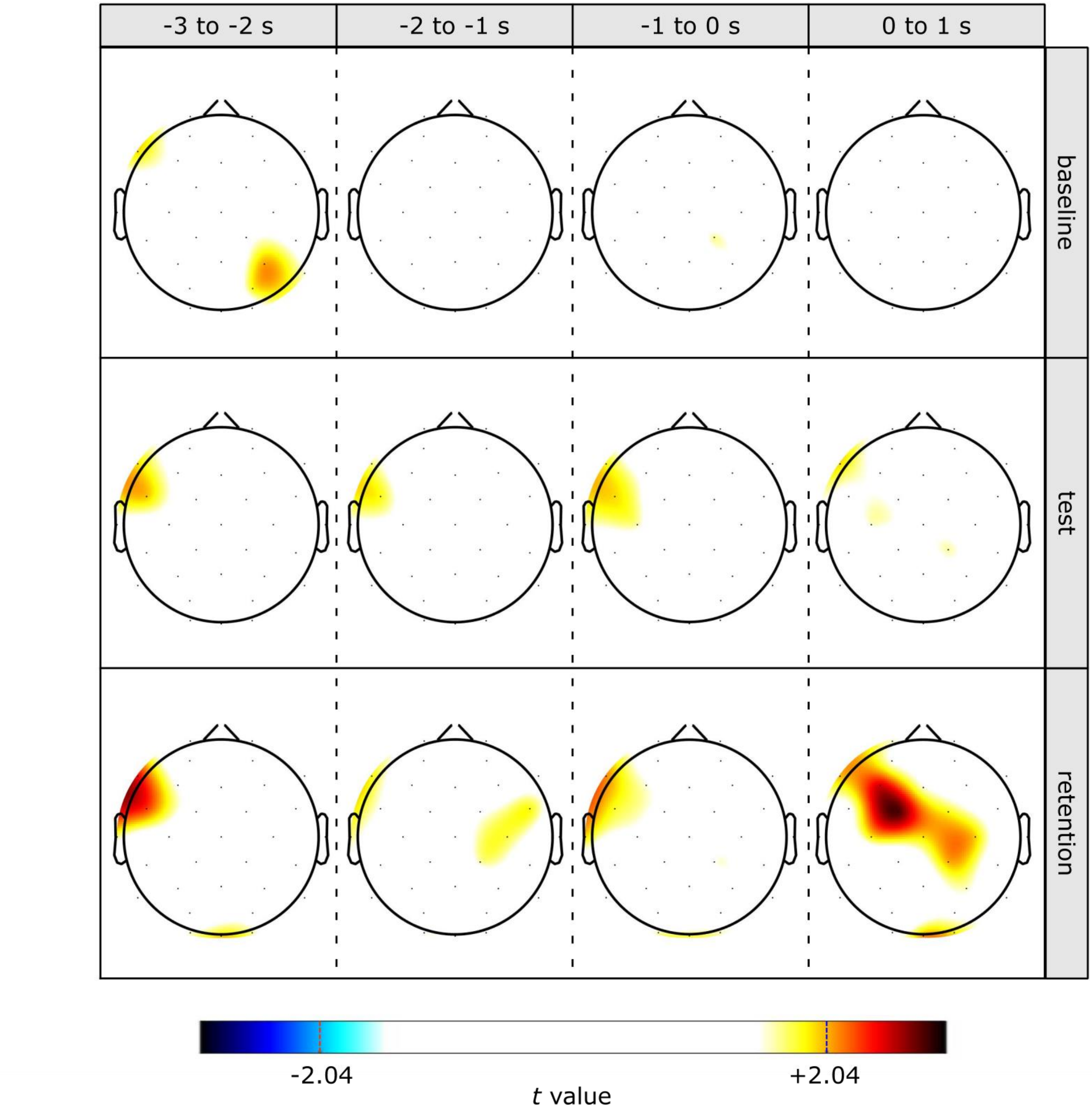

FIGURE A4.11 Replicate of Figure A3.1 (Appendix 3) for the upper alpha band (10-12 Hz).

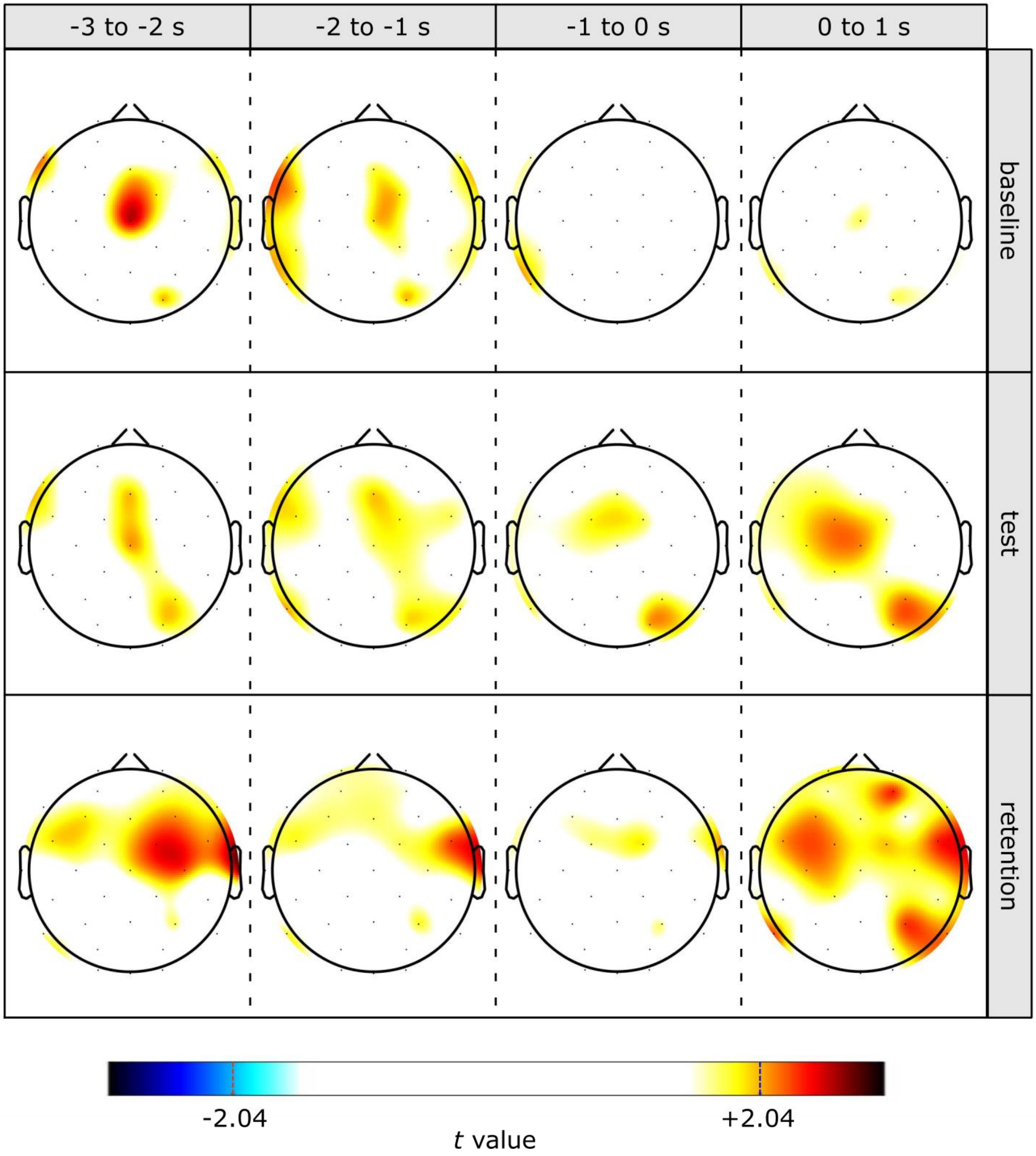

FIGURE A4.12 Replicate of Figure A3.1 (Appendix 3) for the beta band (15-25 Hz).
